# Fus1 KO Mouse As a Model of Oxidative Stress-Mediated Sporadic Alzheimer's Disease: Circadian Disruption and Long-Term Spatial and Olfactory Memory Impairments

**DOI:** 10.3389/fnagi.2016.00268

**Published:** 2016-11-15

**Authors:** Guillermo Coronas-Samano, Keeley L. Baker, Winston J. T. Tan, Alla V. Ivanova, Justus V. Verhagen

**Affiliations:** ^1^The John B. Pierce LaboratoryNew Haven, CT, USA; ^2^Department of Neuroscience, Yale University School of MedicineNew Haven, CT, USA; ^3^Department of Surgery, Yale University School of MedicineNew Haven, CT, USA

**Keywords:** sporadic Alzheimer's Disease, Fus1/Tusc2, Fus1 KO, olfaction, hippocampus, oxidative stress, Morris water maze, olfactory bulb

## Abstract

Insufficient advances in the development of effective therapeutic treatments of sporadic Alzheimer's Disease (sAD) to date are largely due to the lack of sAD-relevant animal models. While the vast majority of models do recapitulate AD's hallmarks of plaques and tangles by virtue of tau and/or beta amyloid overexpression, these models do not reflect the fact that in sAD (unlike familial AD) these genes are not risk factors *per se* and that other mechanisms like oxidative stress, metabolic dysregulation and inflammation play key roles in AD etiology. Here we characterize and propose the Fus1 KO mice that lack a mitochondrial protein Fus1/Tusc2 as a new sAD model. To establish sAD relevance, we assessed sAD related deficits in Fus1 KO and WT adult mice of 4–5 months old, the equivalent human age when the earliest cognitive and olfactory sAD symptoms arise. Fus1 KO mice showed oxidative stress (increased levels of ROS, decreased levels of PRDX1), disruption of metabolic homeostasis (decreased levels of ACC2, increased phosphorylation of AMPK), autophagy (decreased levels of LC3-II), PKC (decreased levels of RACK1) and calcium signaling (decreased levels of Calb2) in the olfactory bulb and/or hippocampus. Mice were behaviorally tested using objective and accurate video tracking (Noldus), in which Fus1 KO mice showed clear deficits in olfactory memory (decreased habituation/cross-habituation in the short and long term), olfactory guided navigation memory (inability to reduce their latency to find the hidden cookie), spatial memory (learning impairments on finding the platform in the Morris water maze) and showed more sleep time during the diurnal cycle. Fus1 KO mice did not show clear deficits in olfactory perception (cross-habituation), association memory (passive avoidance) or in species-typical behavior (nest building) and no increased anxiety (open field, light-dark box) or depression/anhedonia (sucrose preference) at this relatively young age. These neurobehavioral deficits of the Fus1 KO mice at this relatively young age are highly relevant to sAD, making them suitable for effective research on pharmacological targets in the context of early intervention of sAD.

## Introduction

Alzheimer's disease (AD) accounts for more than 80% of dementia incidences and is one of the most common neurodegenerative diseases worldwide (Kumar et al., 2015). AD is a complex disorder that can be subdivided into familial and sporadic cases (Piaceri et al., 2013). Familial AD (fAD) has been associated with mutations in three genes; amyloid precursor protein (APP) (Goate et al., 1991), presenilin 1 (PSEN1) (Sherrington et al., 1995), and presenilin 2 (PSEN2) (Levy-Lahad et al., 1995). fAD predominantly displays between the ages of 35–70 years and is defined as early onset (Ryan and Rossor, 2010). Sporadic AD (sAD), which encompasses ~90% of all AD cases (Anand et al., 2014) is determined by both genetic and environmental factors and usually develops in patients older than 70 years, known as late onset (Bird, 2008). Patients with AD present with progressive mental, behavioral and functional decline. Brain regions involved in the progression of AD include the entorhinal cortex and the hippocampus and correlate with these cognitive changes (Delacourte et al., 1999; Sarazin et al., 2007). Episodic memory is one of the first cognitive functions impaired (Welsh et al., 1991) and remains predominant in the course of the disease and is therefore a core feature for diagnosis. Several studies have also illustrated decreased olfactory function with increasing age symptomatically resulting in difficulty to differentiate odors which can be predictive of broader cognitive deficits such as AD (Doty et al., 1984; Wilson et al., 2009). The involvement of the olfactory system in the initial presentation of AD is a result of damage to central olfactory areas such as the entorhinal cortex and the hippocampus in the early stages of disease (Price et al., 1991; Wilson et al., 2007). As olfactory deficits can appear even prior to clinical symptoms of AD (Masurkar and Devanand, 2014) it has been suggested that testing the olfactory system with respect to accelerated neurodegeneration may offer a unique insight for early detection of cognitive decline (Franks et al., 2015).

Accompanying the widely seen cognitive deficits of AD there is a wide range of non-cognitive behavioral symptoms associated with disease such as anxiety, depression and sleep disturbances (Mega et al., 1996; Musiek et al., 2015). Symptoms of depression can precede the onset of AD (Devanand et al., 1996) and patients with prior diagnosis of depression are more likely to have recurrence during the course of the AD (Strauss and Ogrocki, 1996). Sleep disturbances are common in AD patients with increased wakefulness during the night and increased time spent sleeping during the day. This behavior has been proposed to have important implications on cognition (Hatfield et al., 2004).

Over the last 20 years, many groups have developed drugs in accord with the amyloid cascade hypothesis that target the APP and Aβ peptides. However, this strategy has not been successful. It has been proposed that the deposits of Aβ do not correlate with cognitive impairments and that Aβ deposition can also be found in cognitively normal individuals (Golde et al., 2011; Morris et al., 2014) Some studies have suggested that age is the most accepted risk factor of AD and that age should be the central argument in any hypothesis (Herrup, 2015). Further, other groups have suggested that sAD has not been targeted early enough (Selkoe, 2011).

The “mitochondrial cascade hypothesis,” on the other hand, promotes the idea that the development of sAD is due to age-related mitochondrial energy deficits, increased reactive oxygen species (ROS) production and oxidative stress. The result of these alterations may lead to Aβ accumulation, neuronal death, and dementia (Swerdlow et al., 2010). Indeed, mitochondria-derived ROS themselves can trigger Aβ generation by enhancing the amyloidogenic pathway (Leuner et al., 2012).

It has been suggested that decline of cognitive function in the aging brain can be due to the cumulative presence of ROS. ROS that include superoxide anions, hydrogen peroxide, hydroxyl radicals and hydroxyl anions are highly reactive free radical derivatives of oxygen species that occur as a natural by-product of cellular metabolism, most notably mitochondrial respiration (Milton and Sweeney, 2012). High ROS levels cause oxidative stress that may result in cell death, ultimately leading to neurodegenerative diseases including AD (Lin and Beal, 2006). This link has previously been demonstrated between oxidative damage in the hippocampus of rats and learning impairment (Nicolle et al., 2001). Fus1, a tumor suppressor protein residing in mitochondria, maintains mitochondrial homeostasis and is highly expressed in the brain (Ivanova et al., 2007). Fus1 is involved in regulation of inflammatory and stress responses to various stimuli, infection agents and tumor growth (Hood et al., 2013; Yazlovitskaya et al., 2013, 2015). Fus1 deficient mice display signs of chronic inflammation including increased cytokine production, NF-κB activation and increased levels of ROS (Uzhachenko et al., 2012, 2014). Inflammation leading to ROS production, one of the causative factors of aging, has been shown to reduce expression of Fus1. This reduction however, can be rescued using ROS scavengers demonstrating a ROS-dependent regulation of Fus1 (Ivanova et al., 2009). Therefore, a constant overproduction of ROS due to the aging process may reduce Fus1 expression leading to a state of chronic inflammation (Uzhachenko et al., 2012). In turn, reduced Fus1 expression results in perturbation of mitochondrial homeostasis, exacerbation of ROS production, chronic oxidative stress, which can ultimately lead to sAD as suggested by the “mitochondrial cascade hypothesis' (Swerdlow and Khan, 2004). Thus, Fus1 knockout mouse model may represent a novel model for the study of sAD.

The use of preclinical animal models has been invaluable in the study of AD whereby neuropathological changes, characterized by progressive cognitive decline, ultimately involve multiple cognitive, neuropsychological and behavioral domains. In order to know if the Fus1 knockout female mice display the behavioral and biochemical alterations characteristic for sAD at early stages, we implemented a battery of behavioral tests comparing KO mice with the age- gender- and strain- matched WT mice. We used 4–5 months old mice, the age in which the control mice are considered mature adults but not yet affected by senescence (86% survival at age of 500 days, Anisimov et al., 2015).

Deficits in olfaction can be used as an early indicator of underlying dysfunction and utilizing odor paired-associated tasks are analogous to verbal paired-associated tasks in humans (Bunsey and Eichenbaum, 1996). Here, the habituation/cross-habituation task, which relies upon the animal's tendency to investigate novel odors, tests if the animal can differentiate between the sequential presentation of different odors (Yang and Crawley, 2009). The Morris water maze and passive avoidance tasks are an excellent representation of both learning and memory. Morris water maze is a widely used paradigm to investigate spatial reference learning and memory determined by the animal's ability to learn the location of a hidden platform (Morris, 1984). Meanwhile in the passive avoidance task the animal must refrain from entering a chamber paired with an aversive stimulus, in this case a foot shock, to test associative learning and memory (Hall, 1934; van der Poel, 1967). One final area of investigation included the psychological disturbances associated with aging and AD. These include circadian rhythm disturbances alongside fear and anxiety. The anxiety of the animal model was determined using the open field and the light/dark box to determine freezing, defecation and thigmotaxis (Hall, 1934). Anhedonia, which is the loss of sensitivity to reward and related to depression, was determined by the sucrose preference test (Romano et al., 2015). In light of recently published concerns about reproducibility of animal research (Pusztai et al., 2013; Jilka, 2016) we adopted Noldus video-tracking for all the behavioral assays reported herein. This methodology avoids experimenter bias and increases accuracy of the collected data.

## Experimental procedures

### Subjects

**Figure 1:**
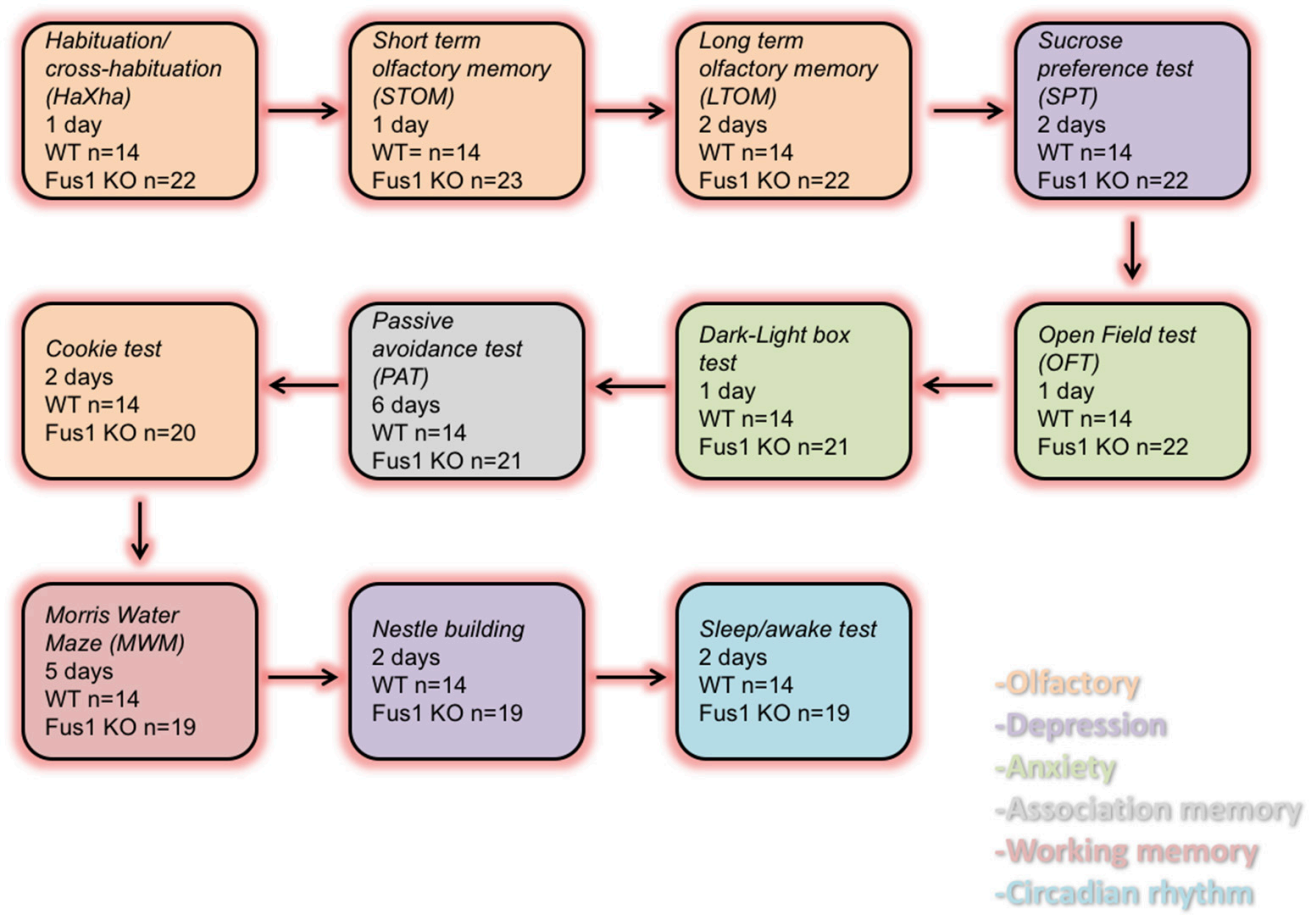
**Diagram representing the order of the behavioral tasks, the number of experimental days and the number of WT and Fus1 KO mice tested**.

In this study we compared the behavior of young female Fus1 KO/129sv mice and Fus1 WT/129sv generated by Dr. Alla Ivanova (Ivanova et al., 2007). We used 5 months old Fus1 KO (*n* = 23) and WT mice (*n* = 14) for the experiments. The vivarium had a 12-h/12-h inverted light cycle with lights off at 10:30 am. All animals were housed individually in polycarbonate cages (12 × 12 × 25 cm) with controlled humidity (40%) and temperature (22°C), and the mice were fed *ad libitum* chow (Harlan 18% protein rodent diet). All the animals were treated according to the guidelines established by the U.S. National Institutes of Health (National Research Council (US) Committee for the Update of the Guide for the Care and Use of Laboratory Animals, 2011). The experimental protocols were approved by the Institutional Animal Care and Use Committee of the John B. Pierce Laboratory. The John B. Pierce Laboratory is AAALAC accredited. Animals were tested as shown in Figure 1.

### Habituation/cross-habituation test (HaXha)

This olfactory-dependent behavioral task was performed in a sealed semi-transparent white acrylic box (26 × 38 × 16 cm) in which a cotton-tipped wood applicator (4 cm long, Puritan REF 806-WC) was presented 1 cm from the bottom of the box and placed on the center, while retained by a 54 × 54 × 4 mm applicator holder. We presented one of the 3 odorants to the applicator: mineral oil (MO, control), amyl acetate (AA) (diluted 1% in MO), phenyl ethanol (PE) (1% in MO), and the social odor (S) (obtained by swabbing the cage of non-experimental CRE-OMP female mice). A total of 12 trials were tested per mouse, where each odorant was presented three times in succession per session to yield the following order: MO1-3, AA1-3, PE1-3, and S1-3. Each trial consisted of 2 min per odorant exposure and inter-trial of 1 min between stimuli. The time spent smelling the cotton tip was registered using a USB camera (Logitech HD Pro C920, 1920 × 1080 pixels) mounted at the ceiling of the box, and used the Noldus behavioral tracking system (EthoVision XT, version 10.1, Noldus Information Technology b.v., Wageningen, The Netherlands) to identify and score the behavior of the animal. Smelling was defined as being oriented toward the applicator tip while the nose is within 2 cm of it. This test evaluates if mice are able to spontaneously recognize a novel odorant stimulus by spending more time smelling the applicator (cross-habituation phase, trial 3 vs. new odor 1), as opposed to the time that the mice spent on each stimulus (habituation phase, trial 1 vs. 3 of same odor).

### Short term and long term olfactory memory (STOM, LTOM)

These behavioral tasks were performed in the same box of the habituation/cross-habituation (HaXha) test, recorded with same camera (Logitech HD Pro C920, 1920 × 1080 pixels). We used Noldus as the software to track the behavior of mice and following the same approach as HaXha. A social odor was used in both behaviors: female urine (obtained from 3 mice and diluted 1% in deionized water) for the short term olfactory memory (STOM), and dam urine (obtained from 3 female dams, 1% in deionized water) for the long term olfactory memory (LTOM). For the STOM, we presented a cotton applicator with female urine (1%) for 20 s after 1 min (3X), 2, 4, 8, and 16 min inter-trial-intervals (ITIs), after exposure of an applicator with only water as a control stimulus on each trial. Therefore, we ensured that the behavioral response across trials was not due to the applicator-diluent presentation, but due to the odorant. We tested LTOM using dam urine (1%) as stimulus for 2 min at 10 min (2X), 30, 60, 120, 240 min to 24 h ITIs, each also preceded by water control presentations.

### Sucrose preference test (SPT)

Our sucrose preference test was based on Romano et al. (2015). The mice were placed in regular home cages (22.5 × 16.7 × 14 cm) and had access to two drinking tubes, one filled with 20 ml tap water, and the other with 20 ml 10% sucrose (Sucrose, S5016, Sigma-Aldrich) diluted in tap water. The location was randomized. During the next 48 h, free consumption of water and 10% sucrose solution took place in the presence of *ad libitum* food. Fluid intake was measured at 24 h intervals. Sucrose preference was calculated from the amount of sucrose solution consumed, expressed as a percentage of the total amount of liquid drunk at each 24-h interval.

### Open field test (OFT)

The Open Field was based on Chen et al. (2013) and Galeano et al. (2014), and was used to assess spontaneous exploratory activity and anxiety-related behaviors (Hall, 1934). Anxiety and exploratory activities were evaluated by allowing mice to freely explore an open field arena for 15 min. The testing apparatus was a classic open field (i.e., a white PVC square arena, 50 × 50 cm, with walls 40 cm high). A video camera (Logitech HD Pro C920, 1920 × 1080 pixels) connected to a Noldus computer system was placed above the box. Each mouse was placed individually on the center of the arena and the performance was monitored by the video tracking system (Noldus System). The central area was arbitrarily defined as a square of 35 × 35 cm (half the total area). The ethological measures analyzed included frequency and duration spent at each area (center and periphery) and locomotor activity.

### Dark-light box test (DLBT)

This task is used to detect activity in disorders related to generalized anxiety and used to complement the OFT (Ramos, 2008). The task is based on the innate aversion of rodents to brightly illuminated areas and on the spontaneous exploratory behavior in response to a novel environment and light (Crawley and Goodwin, 1980; Bourin and Hascoët, 2003). This task was based on Van Dam et al. (2003) and Pinton et al. (2011).

The apparatus consisted of a dual compartment box with free access between them. The dark compartment consisted of gray PVC and had a roof on the top. The other box was exposed and was brightly illuminated by room light. Each animal was placed at the center of the illuminated compartment, facing the central opening to the dark compartment and the time spent in the light compartment was recorded during 5 min. Anxiogenic activity was evaluated by the time spent in the illuminated compartment and the latency for the first light-dark transition.

### Hidden cookie test (HCT)

This test measures the latency of the mice to find a hidden cookie in one of the corners of the home cage. The mice were familiarized with the chocolate cookie for a week before the test. A quarter of chocolate cookie (~2.5 g) was placed every 48 h for three times in the home cage on the top of the bedding. Mice received food and water *ad libitum*. After a week of the familiarization phase, mice were deprived of the cookie for 3 days before the test. On the first day of the test, mice were individually caged with 1 cm of clean bedding. Initially, a piece of cookie was hidden in one corner of the cage and the latency to finding the cookie was recorded and analyzed by Noldus. An hour and a half later, another piece of cookie was placed in the same corner as the first trial, and the behavior was tracked and analyzed. At the end of the second trial, the mice were taken to the animal room. The next day, mice were placed in the experimental cages and the piece of cookie was hidden in a corner different from the last day, and this was tested twice following the same protocol as the first experimental day.

### Morris water maze test (MWMT)

Spatial reference learning and memory were evaluated in a water maze adapted from that previously described by Morris (1984) and was based on Van Dam et al. (2003), Javed et al. (2011), Chen et al. (2013) and Galeano et al. (2014).

The test was performed in a circular galvanized steel pool of 90 cm in diameter and 40 cm height, filled with 20 cm of water tainted with a non-toxic white paint (acrylic paint, 20503 white, Apple Barrel). As it is known that the standard Morris water maze can interfere with physiology, likely due to the stress of heat loss (Iivonen et al., 2003), we opted to refine this method to be less stressful by not maintaining it at room temperature (21 ± 2°C), but instead between room and central body temperature (37°C), i.e., at 29 ± 2°C. The pool was virtually divided into four equal quadrants, labeled north–south–east–west. A camera connected to a Noldus video tracking system was mounted above the maze.

During training, a platform (10 cm in diameter and made of transparent acrylic plastic) was submerged 1 cm below water surface and was placed at a fixed location in one of the quadrants. If the mice were not able to reach the platform within 120 s, they were guided to the platform where they had to stay for 30 s before being returned to their home cage for 30 s. All mice were given four trials per day, once from each quadrant, for 4 consecutive days. The starting position was randomized among four quadrants of the pool. During training trials, the latency to reach the escape platform and the path length were measured.

A probe trial was performed 24 h after the last day of training. During the probe trial, mice were allowed to swim in the pool without the escape platform for 120 s. The latency to reach the platform (s), swim distance (cm), and swim speed (cm/s) were recorded using an automated tracking system (Noldus). During the probe trial, performance was expressed as the percentage of time spent in each quadrant of the MWM, and the number of crossings through the position where the platform used to be during acquisition (using 15 cm diameter).

### Nestlet building task (NBT)

After a 24 h rest period, mice were housed individually and tested for nest building (adapted from Deacon, 2006; Wesson and Wilson, 2011). Two hours prior to the onset of the dark phase of the lighting cycle, individual cages were supplied a commercially available Nestlet pressed cotton square (Ancare, UK agent, Lillico). The next morning (~16 h later) cages were inspected for nest construction. Pictures were taken prior to evaluation for documentation. Nestlet nest construction was scored blind to genotype using the system of Deacon (please see Deacon, 2006, for detailed scoring standard). Briefly, in this 5 point scale, 1 indicates a >90% intact nestlet, whereas a 5 indicates a nestlet torn >90% and a clear nest crater.

### Sleep/awake task

Piezo foil PVDF based circadian rhythm scoring of sleep/wake rhythm was used as established in Donohue et al. (2008), and further used in AD studies (Duncan et al., 2012; Mang et al., 2014; Sethi et al., 2015). Mice were individually housed for 5 days in clean plastic cages (Signal Solutions LLC) with ~5 mm of corn cob bedding lining the floor and lined with the PVDF foil. Activity was recorded for the full duration and analyzed for circadian rhythm of locomotion, REM and NREM sleep.

### Data analysis

Statistical analysis was conducted by GraphPad v6.07. Results were expressed as mean ± standard error (SEM). Repeated measures one-way ANOVA was used for comparison of the same group in the HaXha test (main effect: MO, AA, PE, S; dependent variable: exploration time); as well as the STOM and LTOM tasks (main effect: urine stimuli; dependent variable: exploration time). Two-way ANOVA for SPT [main effects: % preference/mL consumed and groups (WT vs. Fus1 KO)]. For PAT was used a two-way ANOVA (main effects: latency and groups). Two-way ANOVA for OFT (main effects: center/periphery areas and groups; dependent variable: distance traveled, activity, number of fecal boli, zone preference). Repeated measures one-way ANOVA was used for HKT (main effect: cookie stimulus; dependent variable: latency to find the cookie). One-way RM ANOVA for the MWMT (main effect: platform; dependent variable: latency, velocity, distance traveled). All the analyses were followed by Bonferroni-corrected *post-hoc* tests. The rest of the behavioral tests were analyzed using paired and unpaired one-tailed Student's *t*-test. Statistically significant differences were accepted at *P* < 0.05.

To allow for RM ANOVA of behavioral tests with repeated measures we replaced the following missing values with dummy values based on the average for the relevant group and trial: 3 values from the short term memory WT group; 2 values in the swimming velocity MWM (training sessions of the test) for the WT; 1 value in the swimming velocity MWM (training session of the test) for the Fus1 KO; 2 values on the distance traveled MWM (training session of the test) for the WT; 1 value on the distance traveled MWM (training session of the test) for the Fus1 KO.

### Western immunoblot analysis

Protein lysates were prepared from olfactory bulbs and hippocampi of 5 months old WT and KO female mice using RIPA buffer with protease inhibitors followed by sonication by ultrasound and clearing via centrifugation at maximum speed using Eppendorf tabletop centrifuge. Protein concentration was quantified using BCA reagent (Thermo Fisher Scientific, Rockford, IL). Protein extracts (40 μg/well) were subjected to Western immunoblot analysis. We used antibodies against the following proteins: PRDX1 (Sigma), ACC1, pACC1, AMPKα, pAMPKα, NGFR, TrkB, Bcl-xL, Rack1 (all from Cell Signaling Technology), Calretinin (Millipore), LC3 (Abcam). The band intensity was determined using ImageJ software, normalized to loading control (β-actin). The data are presented as a relative intensity of WT bands over KO bands. Phospho-index was determined as a ratio of phosphorylated/total protein intensities.

## Results

### Young female Fus1 KO did not show early olfactory impairments in the habituation task

**Figure 2:**
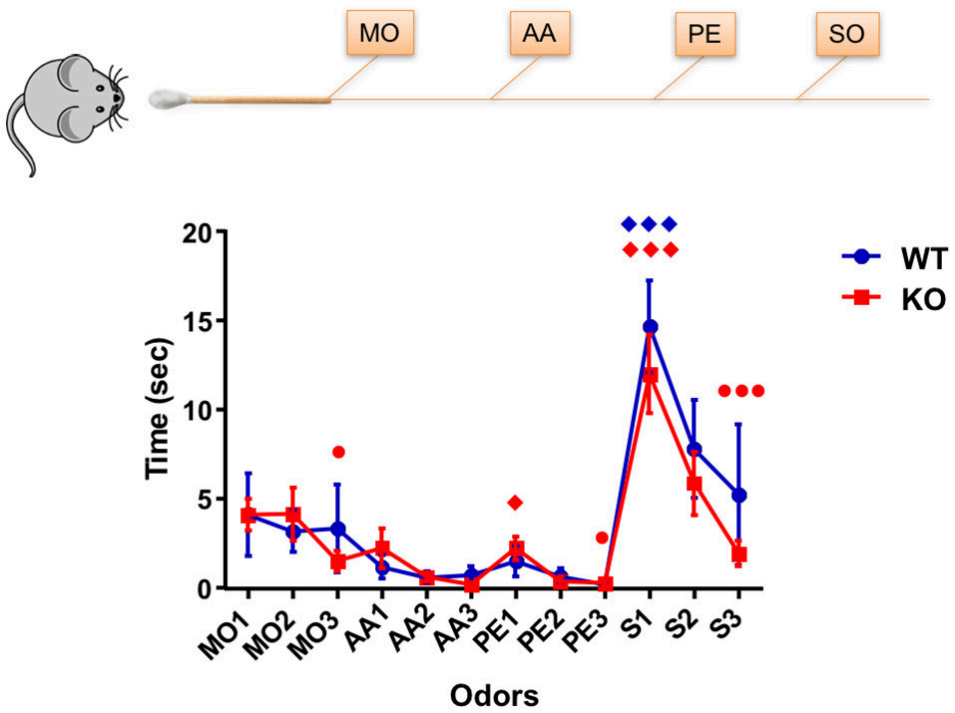
**Habituation/cross-habituation task**. Data depicts mean ± SEM for the time (sec) spent by mice WT (blue; *n* = 14) and Fus1 KO (red; *n* = 22), sniffing within 2 cm of a presented cotton tip with one of the following odors: mineral oil (MO, control), amyl acetate (AA, 1%), phenyl ethanol (PE, 1%), and the social odor (S) (obtained by swabbing the cage of a female mouse. Each odorant was presented three times in succession for 2 min with an inter-trial-interval of 1 min to yield the following order: MO1-3, AA1-3, PE1-3, and S1-3. Significant habituation of the animals to the repeated presentation of an odor is represented as (•) and cross-habituation of an animal to the presentation of an alternate odor is represented as (♦). Habituation was determined at significance difference in time between the first and last presentation of the same odor. Cross-habituation was determined as the significant difference between the last presentation of an odor and the first presentation of an alternate odor. Statistical analysis performed was one-way RM ANOVA with repeated measures followed by Bonferroni's *post-hoc* analysis; ^♦,•^*P* < 0.05, ^♦♦♦,•••^ *P* < 0.001. The Fus1 KO were able to habituate to MO, PE, and S, and cross-habituate for PE and S. The WT mice were able to cross-habituate for S.

Initial experiments using Haxha task established if the young Fus1 KO mice (5 months) showed early signs of olfactory dysfunction when compared to WT animals by evaluating their ability to discriminate between a sequentially presented set of odors. The stimuli used were selected for the following reasons. Mineral oil (MO) was the odorless diluent of the two monomolecular odors and used as a control stimulus, to assess baseline exploration and habituation. Phenyl ethanol (PE) is a non-trigeminal rose-like odorant allowing to selectively probe the olfactory system. Amyl acetate (AA) is an often used banana-like food odorant. Last, we used social odor (SO), as prior work had shown this stimulus to evoke longer exploration times (Coronas-Sámano et al., 2014), thereby enhancing the sensitivity of the test. There was an overall effect on exploration time across the odors [*F*_(2.95, 62.02)_ = 11.68, *P* < 0.001; one-way RM ANOVA, *N* = 22, Figure 2] in the Fus1 KO mice. Firstly, the animals displayed habituation between the repeated presentation of the same odors; MO1 (3.9 ± 0.9 s) and MO3 (1.5 ± 0.6 s; *P* = 0.02), PE1 (2.3 ± 0.7 s) and PE3 (0.3 ± 0.2 s; *P* = 0.03), and for social odor S1 (12 ± 2.2 s) and S3 (1.9 ± 0.7 s; *P* = 0.0007). Secondly, the KO animals displayed cross-habituation between the presentation of different odors; AA3 (0.2 ± 0.1 s) and PE1 (2.3 ± 0.7 s; *P* = 0.02) and PE3 (0.3 ± 0.2 s) vs. S1 (12 ± 2.2 s; *P* = 0.0001). The WT group also showed an effect on the exploration time across stimuli [*F*_(2.31, 30.02)_ = 5.8, *P* = 0.005, *N* = 14, Figure 2]. However, these animals only cross-habituated between PE3 (0.2 ± 0.1 s) and S1 (14.7 ± 2.6 s; *P* = 0.0005). We did not find statistical differences in the exploration time between both groups [*F*_(1, 405)_ = 1.22, *P* = 0.27, two-way ANOVA), nor a significant interaction between both groups and the odor exposures [*F*_(11, 405)_ = 0.5, *P* = 0.89, two-way ANOVA]. We conclude that Fus1 KO mice do not have impaired odor sensitivity (cross-habituation did not differ between groups) or short term odor memory deficits (habituation did not differ between groups).

### WT mice habituated sooner than the Fus1 KO mice in the short term olfactory memory task

Following the basic concept of the HaXha task, but at longer inter-stimulus intervals, we tested the short term olfactory memory in the KO mice. Female urine was used as the stimulus, and the time that each mouse spent with its nose within 2 cm of the cotton applicator, a reasonably accurate estimate of sniffing-mediated odor exploration (Coronas-Samano et al., 2016), was recorded (described in Experimental Procedures).

**Figure 3:**
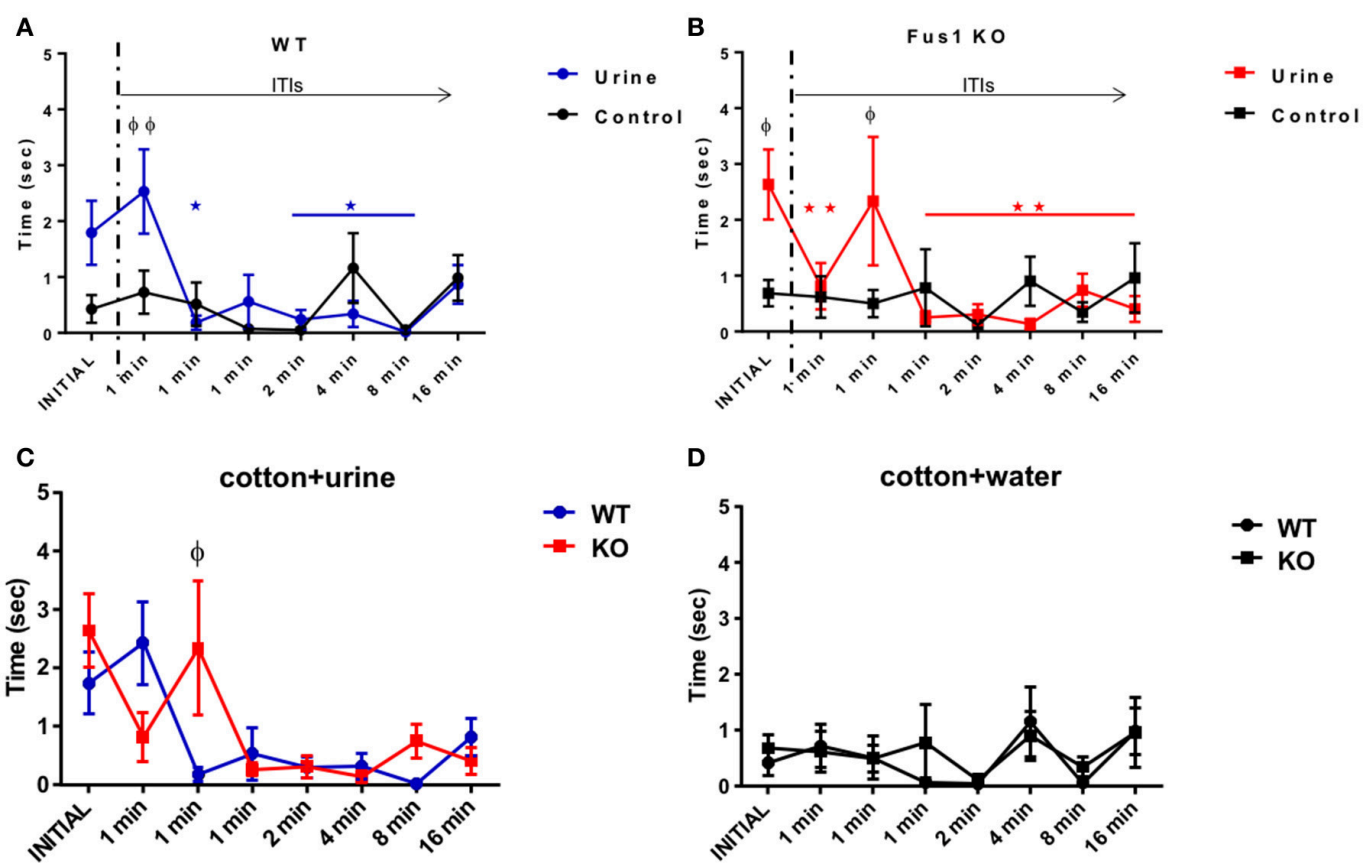
**Short term olfactory memory. (A)** Graphical representation of the time (sec) spent by WT mice (*n* = 14) sniffing within 2 cm of a cotton tip presenting either water (black) or urine (blue) collected from a non-experimental female. Inter-trial-intervals (ITIs) were as follows: 1, 1, 1, 2, 4, 8, 16 min. Habituation (^*^) to the odor was determined by a significant reduction in exploration time when compared to the trial with the highest exploration time (one-way RM ANOVA followed by Bonferroni's *post-hoc, P* <0.05). (φ) denotes significance between the exploration time of the urine and the water control two-way ANOVA followed by Bonferroni's *post-hoc*, ^*^^φ^*P* < 0.05. No significant difference was observed between the initial and last trial for the water control. **(B)** Data illustrates time (sec) spent by Fus1 KO mice (*n* = 23) sniffing within 2 cm of a cotton tip presenting either water (black) or urine (red) collected from a non-experimental female. Habituation determined by a significant reduction in exploration time when compared to the trial with the highest exploration time; one-way RM ANOVA followed by Bonferroni's *post-hoc*, ^*^^φ^*P* < 0.05 was seen by the fourth trial (^*^). Significant difference between exploration time of the urine and the water control is shown in the initial trial (φ) two-way ANOVA followed by Bonferroni's *post-hoc*, ^*^^φ^*P* < 0.05. No significant difference was observed between the initial and last trial for the water control. **(C)** Comparison illustrating the exploration time of the WT (blue; *n* = 14) and Fus1 KO (red; *n* = 23) within 2 cm of a cotton tip presenting urine. Significant difference between groups is denoted as (φ) two way ANOVA followed by Bonferroni's *post-hoc*, ^φ^*P* < 0.05. **(D)** Comparison illustrating the exploration time of the WT (blue; *n* = 14) and Fus1 KO (red; *n* = 23) within 2 cm of a cotton tip presenting water. No statistical differences between groups was observed.

The WT group exploration time was 1.7 ± 0.5 s during the initial exposure of urine and 0.8 ± 0.3 s during the last exposure, trending to reduce their exploration time during the consecutive exposures (*R*^2^ = 0.28, *P* = 0.0003, slope −0.22 s/trial; linear Pearson correlation was used throughout this paper to determine trends). In fact, the WT showed a statistical difference in the exploration time of the urine soaked cotton tip [*F*_(2.34, 30.45)_ = 5.06, *P* = 0.009, one-way RM ANOVA, *N* = 14, Figure 3A, line in blue]. The WT mice habituated to the urine odor from the third exposure, showing statistical differences from the initial exposure 1.7 ± 0.5 s compared with 1 min ITI (third exposure; 0.2 ± 0.1 s, *P* = 0.03), similarly after 2 min ITI (0.3 ± 0.2 s, *P* = 0.03), after 4 min ITI (sixth exposure; 0.3 ± 0.2 s, *P* = 0.02), after 8 min ITI (seventh exposure; 0.01 ± 0.01 s, *P* = 0.03). The WT mice did not show statistical differences in the fourth exposure (0.5 ± 0.4 s, *P* = 0.95) nor the last exposure (0.8 ± 0.3 s, *P* = 0.69). The WT group did not show any effect in the exploration time across the trials [*F*_(2.48, 32.23)_ = 1.86, *P* = 0.16, one-way RM ANOVA, *N* = 14, Figure 3A, line in black) when investigating the water control. In fact, they explored 0.4 ± 0.2 s in the initial trial and 0.9 ± 0.4 s during the last trial, with no trend over trials (*R*^2^ = 0.13, *P* = 0.55). Comparisons between the exploration time of the presented cotton tips with water or urine stimuli illustrated a difference between responses [*F*_(7, 197)_ = 2.78, *P* = 0.009; two-way ANOVA, Figure 3A]. This was evident by the increase in exploration time observed between the second exposure (after 1 min ITI) of water (0.7 ± 0.4 s) vs. urine (2.4 ± 0.7 s; *P* = 0.005).

Fus1 KO mice also showed changes in exploration time with repeated urine exposures [*F*_(1.78, 39.1)_ = 4.59, *P* = 0.01 one-way RM ANOVA, *N* = 23, Figure 3B, line in red]. The KO mice habituated on the second trial. The above was evident when comparing the time spent exploring the initial exposure (2.6 ± 0.6 s) with the 1 min ITI (second trial; 0.8 ± 0.4 s, *P* = 0.001), 1 min ITI (fourth trial; 0.3 ± 0.1 s, *P* = 0.006), 2 min ITI (fifth trial; 0.3 ± 0.1 s, *P* = 0.005), 4 min ITI (sixth trial; 0.13 ± 0.09 s, *P* = 0.002), 8 min ITI (seventh trial; 0.7 ± 0.2 s, *P* = 0.04), 16 min ITI (eight trial; 0.4 ± 0.2 s, *P* = 0.004). The Fus1 showed “cross habituation” (i.e., reversal of habituation to the same stimulus) by the third exposure which did not show a statistical difference to the initial odor exploration time (1 min ITI; 2.3 ± 0.4, *P* > 0.99). In comparison, these animals did not show a significant change in exploration time when presented with cotton soaked with water [*F*_(2.38, 52.39)_ = 0.62, *P* = 0.57, one-way RM ANOVA, *N* = 23, Figure 3B, line in black], showing lower responses of 0.7 ± 0.2 s on the initial exposure to 0.9 ± 0.6 s on the last trial with no trend across trials (*R*^2^ = 0.02, *P* = 0.81). Further, the comparisons between the cotton presentation of water or urine stimuli demonstrate significant [F_(7, 308)_ = 3.23, *P* = 0.003, two-way ANOVA, *N* = 23, Figure 3B] differences between the initial exposure of water (0.7 ± 0.2 s) vs. urine (2.6 ± 0.6 s) (*P* = 0.03) a difference also seen in the third exposure to water 0.5 ± 0.2 s vs. urine 2.3 ± 1.2 s (*P* = 0.04).

The WT and Fus1 KO groups did not show differences in urine exploration [*F*_(1, 278)_ = 0.43, *P* = 0.51; two-way ANOVA, Figure 3C]. Nevertheless, the interaction between groups and exposures was significant [*F*_(7, 278)_ = 2.32, *P* = 0.02; two-way ANOVA Figure 3C], reflecting their different temporal profiles. Fus1 KO showed higher exploration time during the third exposure (after 1 min ITI) 2.3 ± 1.2 s to the stimulus compared with 0.17 ± 0.1 s from the WT (*P* = 0.02). Finally, there was no difference between the WT and Fus1 KO groups related to the amount of time spend exploring the cotton tip with the water stimulus [*F*_(1, 279)_ = 0.3, *P* = 0.58; two-way ANOVA, Figure 3D], nor the interaction effect between groups throughout the trials [*F*_(7, 279)_ = 0.26, *P* = 0.97; two-way ANOVA]. The relatively late habituation by Fus1 KO mice may indicate a mild impairment of short-term odor memory in the Fus1 KO mice.

### Fus1 KO mice did not habituate in the long term olfactory memory task

**Figure 4:**
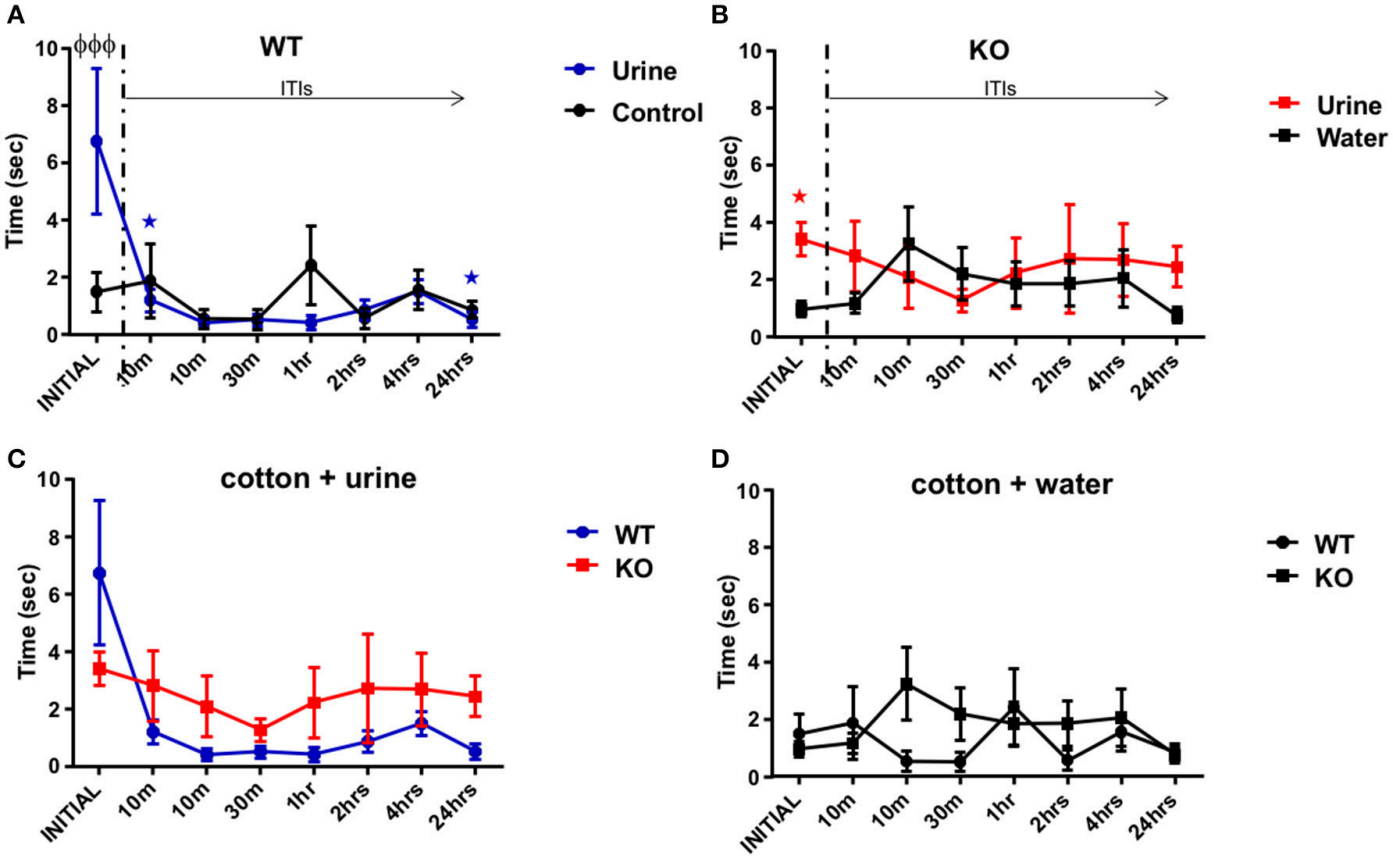
**Long term olfactory memory. (A)** Illustration of the time (sec) spent by WT mice (*n* = 14) sniffing within 2 cm of a cotton tip presenting either water (black) or urine (blue) collected from a non-experimental dam female. Inter-trial-intervals (ITIs) were as follows: 10, 10, 30 min, 1, 2, 4, 24 h. A planned comparison was carried out to know if the exploration time decreased after the initial stimulus of urine. The WT group decreased their time smelling the urine from the initial to the second exposures, also at the end of the task 24 h later (paired one-tailed Student's *t*-test, ^*^P < 0.01) Further, there was a significant difference in the exploration time at the initial trial between urine and water (two-way ANOVA followed by Bonferroni's *post-hoc*, ^φ^*P* < 0.001). **(B)** Data illustrates time (sec) spent by Fus1 KO mice (*n* = 22) sniffing within 2 cm of a cotton tip presenting either water (black) or urine (red) collected from a non-experimental dam female. No significant differences were observed between exploration time of the urine and the water control or between the initial and later trials. For the initial trial Fus1 KO mice explored the urine more than the water control (^*^P < 0.0001, planned one-tailed paired *t*-test). **(C)** Comparison illustrating the exploration time of the WT (blue; *n* = 14) and Fus1 KO (red; *n* = 22) within 2 cm of a cotton tip presenting urine. No significant difference between groups was observed. **(D)** Comparison illustrating the exploration time of the WT (blue; *n* = 14) and Fus1 KO (red; *n* = 22) within 2 cm of a cotton tip presenting water. No statistical differences between groups was observed.

The aim of this behavioral task was to elucidate whether the animals were able to habituate to repeated presentations of the same stimulus (dam urine, in contrast to non-pregnant female urine in the STM) over minutes to hours. The WT group showed statistical differences in the effect of exploration time observed in response to the urine stimulus over repeated exposures [*F*_(1.19, 15.52)_ = 5.31, *P* = 0.03; one-way RM ANOVA, *N* = 14, Figure 4A, blue line], although we did not find statistical differences comparing the initial exposure to the subsequent seven exposures over time *post-hoc*. However, the WT group reduced their urine exploration time from 6.8 ± 2.5 s at the initial exposure to 0.5 ± 0.3 s after 24 h ITI (*R*^2^ = 0.29, *P* = 0.001 slope −0.49 s/trial). A planned comparison between the initial and the second exposures (after 10 min ITI) was significant (one-tailed paired *t*-test *P* = 0.02), suggesting that the WT were able to habituate to the urine after a long inter trial period, even at the end of the task 24 h later (one-tailed paired *t*-test *P* = 0.02). As we expected from the STM task, the cotton soaked with only water (the control stimulus) did not represent a stimulus of interest for the WT group, since they did not show any trend in exploration time, from the initial exposure to the cotton tip (1.5 ± 0.7 s) to the exposure 24 h later ITI [0.9 ± 0.3 s; *R*^2^ = 0.058, *P* = 0.7; *F*_(2.96, 38.52)_ = 0.81, *P* = 0.5, one-way RM ANOVA, *N* = 14, Figure 4A, black line]. In addition, WT mice showed significant differences in exploration time on the initial presentation of urine (6.8 ± 2.5 s vs. water 1.5 ± 0.7 s; *P* < 0.0001, Figure 4A).

The Fus1 KO mice did not show any changes in exploration time for the urine stimulus [*F*_(2.89, 60.73)_ = 0.37, *P* = 0.76, *N* = 22, Figure 4B, red line]. They did not tend to reduce their exploration time across trials (*R*^2^ = 0.02, *P* = 0.73), and a planned comparison did not reveal a difference in exploration time between the initial trial (3.4 ± 0.6 s) and the second exposure (2.8 ± 1.2 s; one-tailed paired Student's *t*-test *P* = 0.15), or even 24 h later (2.4 ± 0.71 s; one-tailed paired Student's *t*-test *P* = 0.15), suggesting that they did not habituate to the urine stimuli. This was tentatively substantiated by the finding that the Fus1 KO mice did explore the urine (3.4 ± 0.58 s) more than the water control (0.95 ± 0.27; *P* < 0.001, planned paired one-tailed *t*-test). Also, they did not show changes in the exploration time for the control stimulus (cotton tip with water) over time [*F*_(3.77, 79.23)_ = 1.18, *P* = 0.33; one-way ordinary RM ANOVA, Figure 4B, black line] and they did not show a trend across trials from the initial exposure (1.0 ± 0.3 s) to the last (0.8 ± 0.3 s; *R*^2^ = 0.05, *P* = 0.88). Further, the Fus1 did not show statistical differences between the exploration time of the control and dam's urine stimuli [*F*_(7, 294)_ = 0.99, *P* = 0.43].

We did not find any differences between the WT and Fus1 KO groups exploration time in response to the urine stimulus [*F*_(1, 272)_ = 2.67, *P* = 0.1; two-way ANOVA, Figure 4C), nor a significant interaction between groups and trials [*F*_(7, 272)_ = 1.2, *P* = 0.29; two-way ANOVA]. The groups did not show any differences in exploration time for the water control [*F*_(1, 272)_ = 1.16, *P* = 0.2; two-way ANOVA, Figure 4D], nor significant interaction between groups and exposures [*F*_(7, 272)_ = 1.14, *P* = 0.34; two-way ANOVA]. Overall, the lack of habituation of the Fus1 KO mice may suggest they have mold long term odor memory impairments.

### Young Fus1 KO female mice do not show anhedonia

**Figure 5:**
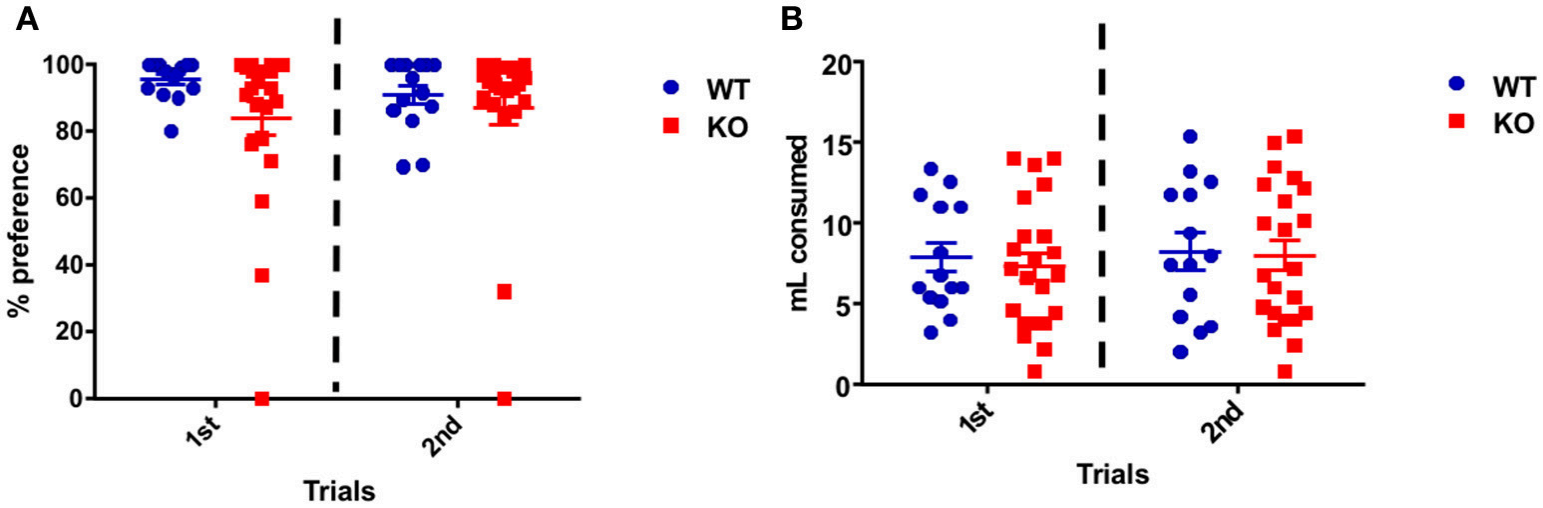
**Sucrose preference task. (A)** Percentage preference dot plot representing individual animal consumption of sucrose (10%) in water when compared to the water control. Sucrose consumption is expressed as a percentage of overall consumption of both sucrose and control for the WT (*n* = 14; blue) and Fus1 KO (*n* = 22; red) mice over two experimental days. Data is averaged for all animals for overall preference of mean ± SEM. No difference in the total sucrose preference was observed between both groups. **(B)** Dot plot representing the overall individual animal consumption (mL) of sucrose (10%) in water and the water control for the WT (*n* = 14; blue) and Fus1 KO (*n* = 22; red) mice over two experimental days. Data is averaged for all animals for liquid consumption (mean ± SEM). No statistical difference in liquid consumption was observed between groups.

On the first day of the test both groups showed high sucrose preference expressed as the percentage sucrose ingested over the total amount ingested: WT 96.6 ± 1.5% and Fus1 KO 84.1 ± 5.2%. This preference was maintained during the second day of the test, in which WT consumption was 90.9 ± 2.9% and the KO 87.2 ± 5.1% with no statistical difference between groups [*F*_(1, 68)_ = 2.55, *P* = 0.12; two-way ANOVA, WT *N* = 14, Fus1 KO *N* = 22, Figure 5A].

Also the total amount of water and 10% sucrose consumed was measured for every day of the test. Over the first day, the WT mice consumed 7.9 ± 0.9 mL and the Fus1 KO 7.3 ± 0.8 mL. Similarly, on the second day, the WT consumed 8.3 ± 1.1 mL and the KO 8.0 ± 0.9 mL with no differences between groups [*F*_(1, 68)_ = 0.02, *P* = 0.87; two-way ANOVA, Figure 5B]. We conclude that Fus1 KO mice did not show anhedonia, a measure of depression.

### Fus1 KO mice have no locomotor or anxiety alterations in the open field test

This test evaluates the locomotor function of mice by collecting data regarding their velocity, total distance traveled, number of fecal boli as a sign of emotional stress (Hall, 1934; Archer, 1973), level of anxiety (total time in the periphery-center areas) and locomotor activity (active or inactive) of every mouse.

**Figure 6:**
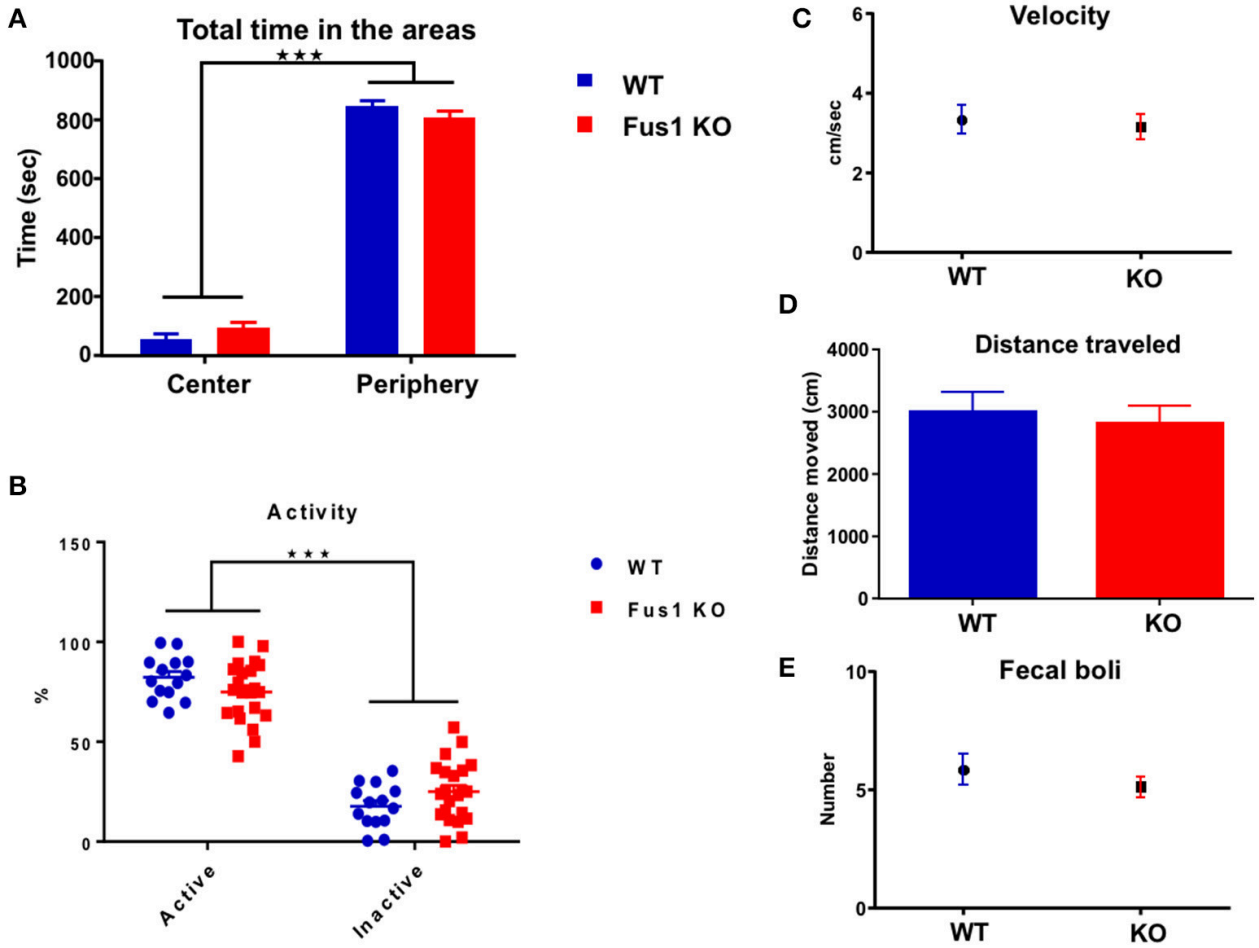
**The open field test. (A)** Bar chart illustrating the total exploration time (sec; mean ± SEM) of WT (blue; *n* = 14) and Fus1 KO (red; *n* = 20) animals in either the center or the periphery of the experimental box. Significance determined by two way ANOVA followed by Bonferroni's *post-hoc*, ^***^P < 0.001. **(B)** Percentage dot plot of activity of WT (blue; *n* = 14) and KO (red; *n* = 20) over the duration of the task (15 min). Average activity and inactivity were determined as a percentage of total movement (mean ± SEM). Significance was obtained by two way ANOVA followed by Bonferroni's *post-hoc*, ^***^P < 0.001. **(C)** Movement velocity (cm/sec) was calculated (mean ± SEM) over trials during the OFT (15 min) for WT (blue; *n* = 14) and KO (red; *n* = 20). **(D)** Bar chart representing the distance traveled (cm) by WT (blue; *n* = 14) and KO (red; *n* = 20) during the OFT. **(E)** Number of fecal boli (mean ± SEM) counted at the end of the trials. No statistical difference was observed for the velocity, distance traveled, or fecal boli.

The arena field was divided in two areas, center and periphery, by configuring the Noldus behavioral tracking. The total time in which every animal spent in each area was measured. The WT and Fus1 KO mice showed statistical differences in the total time spent exploring each arena zone [*F*_(1, 68)_ = 1133, *P* < 0.001; two-way ANOVA, WT = 14, Fus1 = 22, Figure 6A], however, both groups spent more time in the periphery (WT = 846 ± 20 s; Fus1 KO = 808 ± 22 s) rather than on the center of the cage (WT = 53 ± 20 s; Fus1 KO = 92 ± 22 s; *P* < 0.001).

Further, the percentage of time in which animals were active vs. inactive was determined. Both WT and KO mice showed statistical difference between their activity and inactivity periods [*F*_(1, 68)_ = 306.3, *P* ≤ 0.001; two-way ANOVA, Figure 6B]. The WT and the Fus1 KO showed a higher percentage of activity compared to inactivity (WT 82.3 ± 2.9% active, 17.8 ± 2.9% inactive, *P* < 0.001; Fus1 KO 74.9 ± 3.2 active, 25 ± 3.2% inactive, *P* < 0.0001) and there were no differences between groups relating to percentage of activity (*P* = 0.7).

We measured velocity and the total distance traveled of every mouse during the test to establish whether any potential differences between the groups on the main test outcome could be explained thereby and also to establish if any were motorically impaired. The velocity of the WT during the task was 3.3 ± 0.4 cm/s, while the Fus1 KO obtained 3.2 ± 0.3 cm/s, not showing statistical differences (one-tailed unpaired Student's *t*-test, *P* = 0.36, Figure 6C). The WT traveled 3000 ± 316 cm and the Fus1 KO 2813 ± 288 cm with no statistical differences between groups (one-tailed unpaired Student's *t*-test *P* = 0.34, Figure 6D).

In addition, the total number of fecal boli was counted at the end of every trial and compared between groups. The defecation in rodents has been considered a reliable index of emotionality and reflects the level of activation of its sympathetic nervous system when they respond to a different stress situations (Seliger, 1978). No differences were observed in the total fecal boli counts (WT 6 ± 1 boli; Fus1 KO 5 ± 0.4 boli, one-tailed unpaired Student's *t*-test, *P* = 0.17, Figure 6E). These data suggest that Fus1 KO mice did not show increased anxiety over WT.

### The latency to cross to the dark box was longer in the Fus1 KO mice than in the WT mice

**Figure 7:**
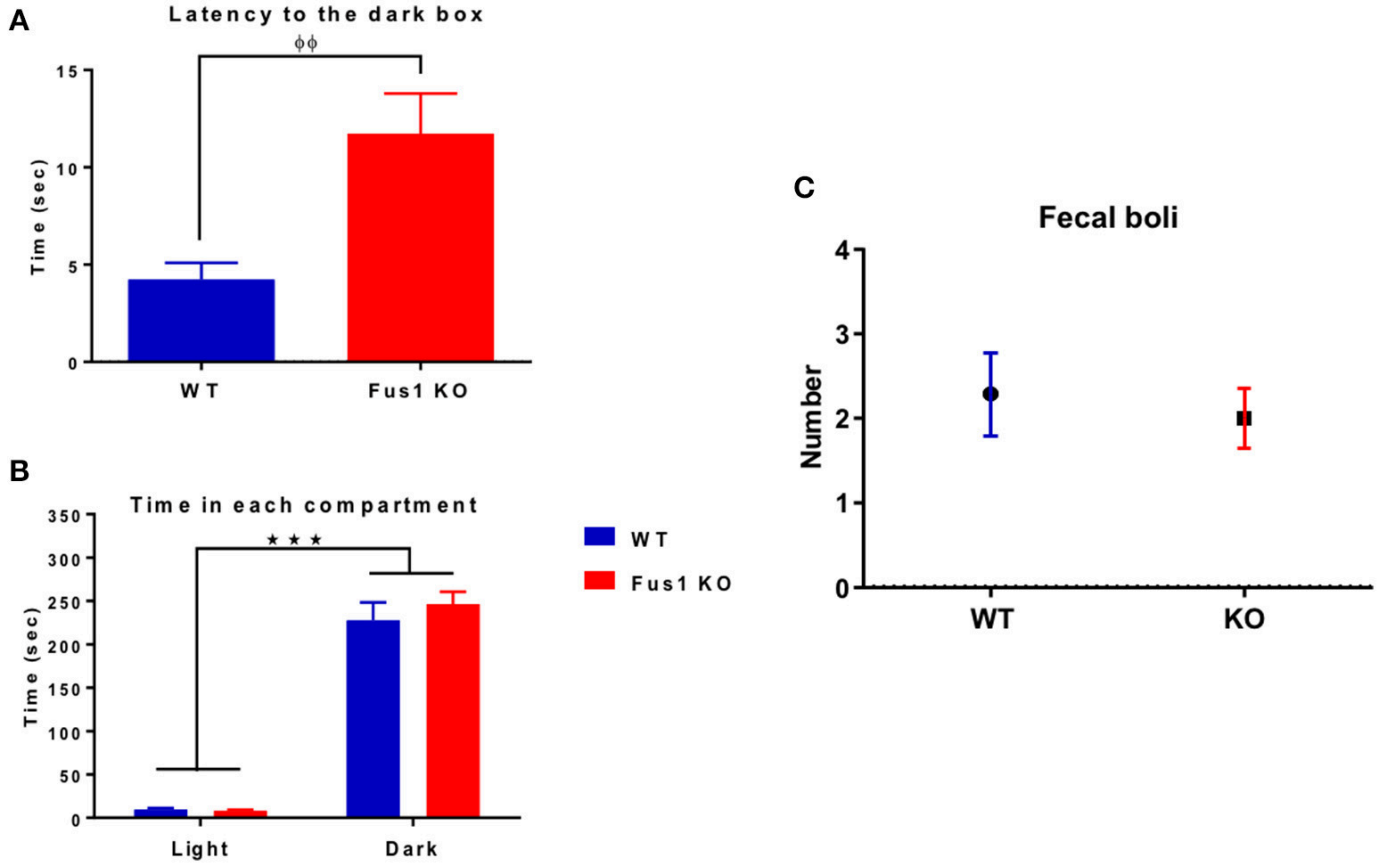
**The dark-light box test. (A)** Bar chart illustrating the initial latency (in seconds) of WT (blue; *n* = 14) and Fus1 1 KO (red; *n* = 21) groups to cross from the light box to the dark box. Significant latency group difference denoted by (φ) unpaired one-tailed students *t*-test, *P* < 0.01. **(B)** Bar chart illustrating total time spent (sec, mean ± sem) by WT (blue; *n* = 14) and Fus1 1 KO (red; *n* = 21) in the light and dark box. Both groups spent more time (and each of similar amount) in the dark than in the light box (two way ANOVA, ^***^P < 0.001). **(C)** Number of fecal boli (mean ± SEM) counted at the end of the trials.

The main objective of this task is to evaluate the latency of every mouse to cross from the light to the dark box. In the case of the WT group (*n* = 14), they spent 4.2 ± 0.9 s crossing from the light to the dark box. Interestingly, the Fus1 KO mice (*n* = 21) spent 11.8 ± 2.1 s, an increase in latency in comparison to the WT group (one-tailed unpaired Student's *t*-test *P* = 0.003, Figure 7A).

Both the WT and Fus1 KO spent more time in the dark than in the light boxes (Figure 7B; WT 228 ± 21 s in the dark, 9.4 ± 1.8 s in the light, *P* < 0.001; Fus1 KO 246 ± 14 s in the dark, 8 ± 1.1 s in the light, *P* < 0.001). No differences were observed between groups [*F*_(1, 66)_ = 0.69, *P* = 0.4, two-way ANOVA] in the total time spent in the light or the dark boxes (*P* > 0.99).

Finally, the number of fecal boli was counted for each group at the end of the test, no differences were observed between WT (2.3 ± 0.5 boli) and the Fus1 KO (2.0 ± 0.4 boli; one-tailed unpaired Student's *t*-test *P* = 0.32, Figure 7C). We conclude that anxiety levels did not differ between the groups as the total time in the dark and the number of boli was the same, despite a difference in latency (which is generally considered a less robust anxiety measure than total time).

### Fus1 KO mice were able to learn the passive avoidance test

**Figure 8:**
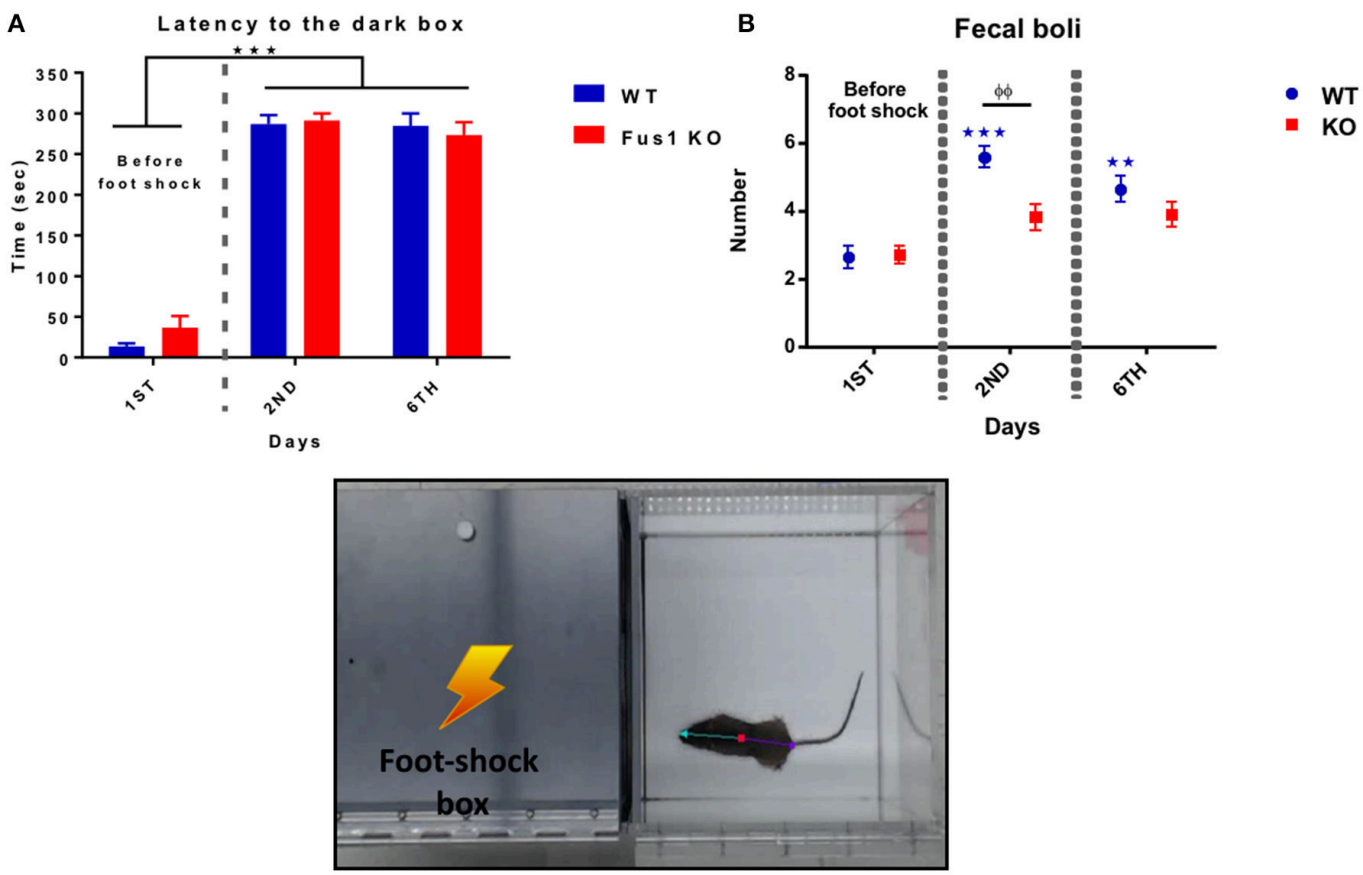
**Passive avoidance test. (A)** Shows the latency (sec, mean ± SEM) of crossing from the light to the dark box prior to a foot shock (Day 1) and post-foot shock (day 2 and 6) by the WT (blue) and Fus1 KO (red). Significant difference between latency of day 2 and 6 compared with day 1 was performed using one way RM ANOVA followed by Bonferroni's *post-hoc*, ^***^P < 0.001. **(B)** Differences in the number of fecal boli (mean ± SEM) on the first day (before the foot shock) compared with the second and sixth day of the test (after foot shock) for the WT (blue) and Fus1 KO (red). Two way ANOVA followed by Bonferroni's *post-hoc*, ^φφ,^^**^P < 0.01, ^***^P < 0.001, was performed between groups.

In this test, mice learn to avoid an environment in which an aversive stimulus (foot-shock) was previously delivered (dark compartment). During this behavioral test, the WT mice illustrated a statistical difference in latency crossing from the light to the dark compartment over the three experimental days after foot-shock, increasing their time from the first trial to the last [*F*_(1.49, 19.32)_ = 197.4, *P* < 0.0001, one-way RM ANOVA, *N* = 14, Figure 8A], showing differences in latency from the first day (14 ± 4 s) compared to the second day (287 ± 11 s, *P* < 0.0001) and the sixth day (285 ± 16 s, *P* < 0.0001).

The Fus1 KO mice also demonstrated changes in latency, increasing their time from the first to the last days [*F*_(1.91, 38.31)_ = 129.1, *P* < 0.0001, one-way RM ANOVA, *N* = 21, Figure 8A] and showing differences from the first trial (36 ± 14 s) compared with the second day (291 ± 9 s; *P* < 0.0001) and the sixth day (274 ± 16; *P* < 0.0001). We did not find differences in the latency between the groups [*F*_(1, 98)_ = 0.256, *P* = 0.61, two-way ANOVA].

This test also measures emotional stress by way of the number of fecal boli after every trial. The WT group showed an increase in the number of fecal boli over the trials [*F*_(1.49, 19.31)_ = 23.55, *P* < 0.0001, one-way RM ANOVA, *N* = 14, Figure 8B] with statistical differences on the first trial (2.6 ± 0.3 boli) compared to the second trial (5.5 ± 0.3 fecal boli; *P* < 0.0001), and the third trial (4.6 ± 0.3 fecal boli; *P* = 0.0004).

The Fus1 KO also did not show an increase in the number of fecal boli over the trials [*F*_(1.58, 31.58)_ = 3.42, *P* = 0.06, one-way RM ANOVA, *N* = 21, Figure 8B]. We found differences in boli between groups [*F*_(1, 98)_ = 7.94, *P* = 0.006, two-way ANOVA, Figure 8B] in the second day of the task (*P* = 0.002). We conclude that as latencies did not differ, there was no difference in associative memory between the groups. Meanwhile, in this test Fus1 KO mice may have been less anxious than WT.

### Fus1 KO mice show impairment in the cookie test

**Figure 9:**
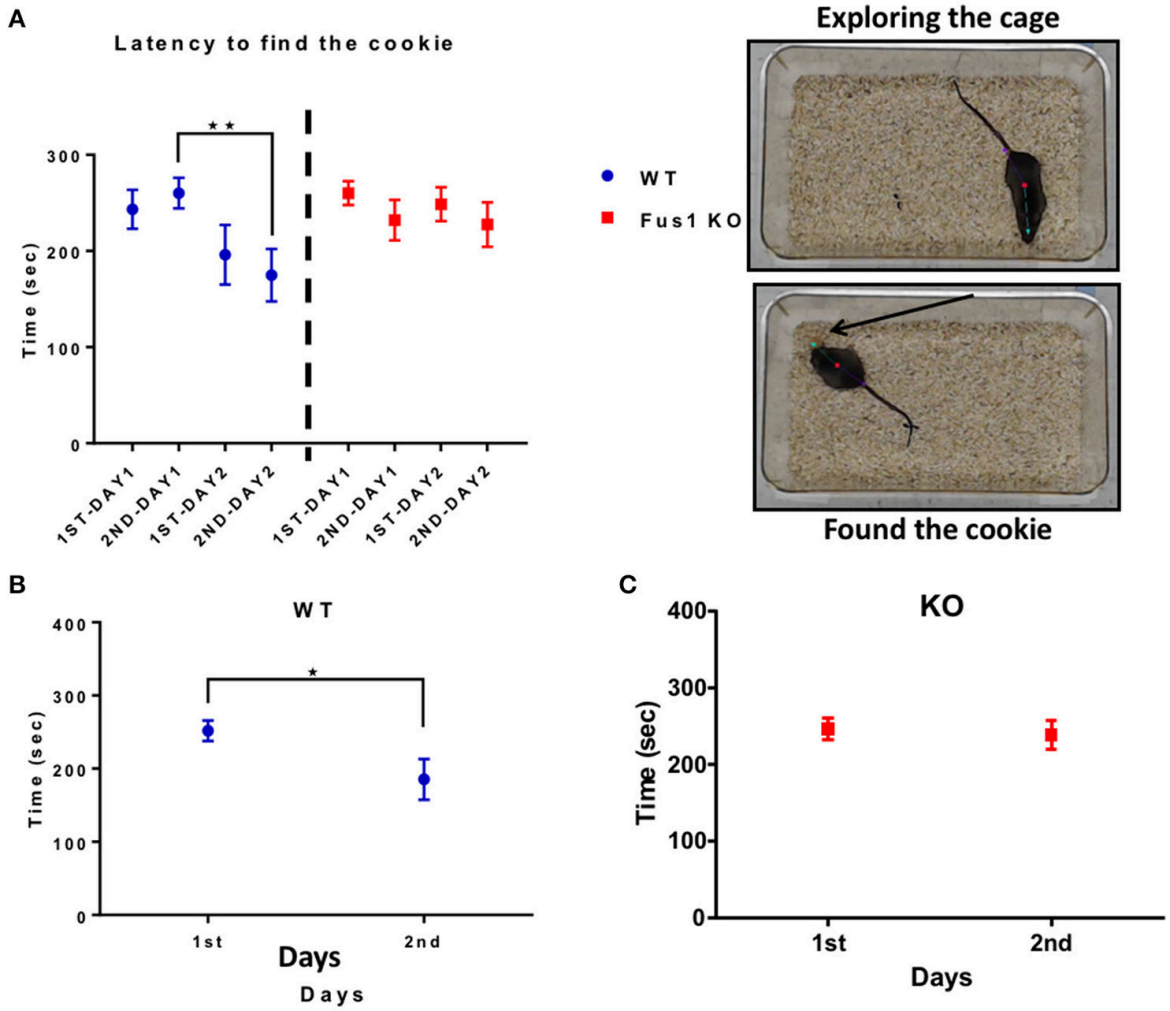
**The hidden cookie test. (A)** Illustrates the latency (sec; mean ± SEM) of the WT blue; *n* = 14 and Fus1 KO (red; *n* = 20) to find the hidden piece of cookie buried within bedding over two consecutive days 2 trials per day. One way RM ANOVA followed by Bonferroni's *post-hoc*, ^**^P < 0.01. **(B)** The average latency (sec) of trials compared over 2 days for WT animals. Paired one-tailed students *t*-test, ^*^P < 0.05. **(C)** The average latency (sec) of trials compared over 2 days for Fus1 1 KO animals.

This test measures how fast the animals can find a familiar piece of chocolate cookie that is hidden in a corner underneath a layer of bedding, but in different locations between the 2 days. The WT group showed a difference in the latency to finding the cookie over 2 days [2 trials/day; *R*^2^ = 0.1, *P* = 0.01, slope −13.5 s/trial; *F*_(2.0, 26.04)_ = 5.2, *P* = 0.01, RM one-way ANOVA, *N* = 14, Figure 9A]. The time was significantly reduced on the second trial of the day 1 (260 ± 16 s) compared to the second trial of the day 2 (175 ± 27 s, *P* = 0.003). Further, the intra-day averages in latency between day 1 (252 ± 14 s) and day 2 (186 ± 28 s) were significant (one-tailed paired *t*-test *P* = 0.006, Figure 9B).

In contrast, the Fus1 KO mice did not show statistical difference in the latency over trials [*R*^2^ = 0.01, *P* = 0.34, *F*_(2.5, 47.51)_ = 1.137, *P* = 0.34, RM one-way ANOVA, *N* = 20, Figure 9A]. Additionally, the intra-day averages of latency from day 1 (246 ± 15 s) compared to day 2 (238 ± 19 s) were not significant (one-tailed paired Student's *t*-test *P* = 0.32, Figure 9C). In conclusion, Fus1 KO mice did not improve their odor guided foraging, in contrast to WT mice.

### Fus1 KO mice showed a low performance in the training sessions of the morris water maze compared to the WT mice

**Figure 10:**
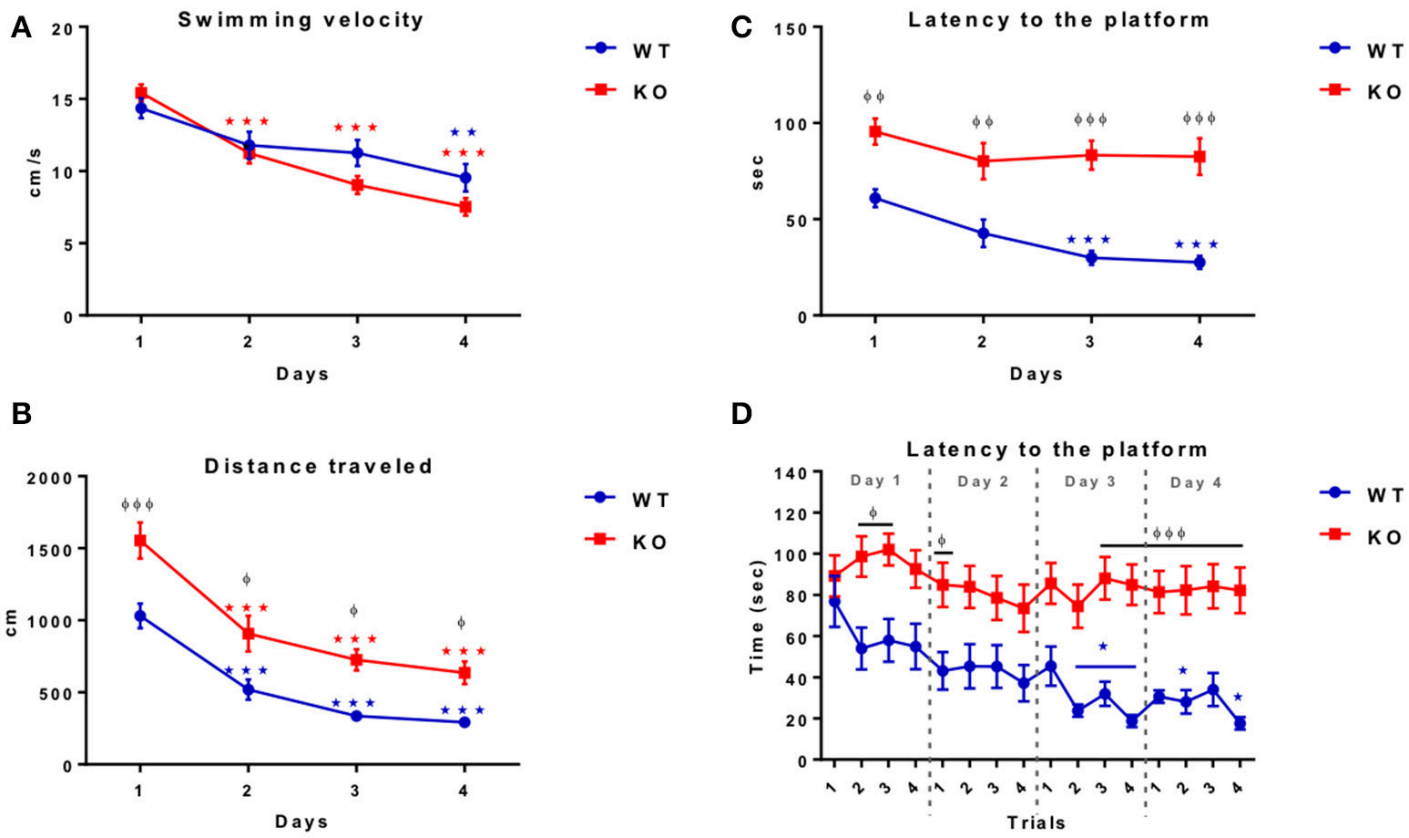
**Morris water maze training. (A)** Shows the swimming velocity (cm/sec) of WT (blue; *n* = 14) and Fus1 1 KO (red; *n* = 19) over 4 days of training. Significance is determined by a change in velocity compared with day 1. One way RM ANOVA followed by Bonferroni's *post-hoc*, ^*^P < 0.05, ^**^P < 0.01, ^***^P < 0.001. Both WT and KO decreased their swimming velocity. **(B)** Illustrates the swimming distance (cm) of WT (blue; *n* = 14) and Fus1 1 KO (red; *n* = 19) over 4 days of training. Significance is determined by a change in velocity compared with day 1. One way RM ANOVA followed by Bonferroni's *post-hoc*, ^***^P < 0.001. Differences between groups shown as ^φ^*P* < 0.05, ^φφφ^*P* < 0.001, two way ANOVA. Both groups decreased their distance however, overall Fus1 KO traveled further across training days. **(C)** Demonstrates the latency to find the hidden platform by the WT (blue; *n* = 14) and Fus1 1 KO (red; *n* = 19) over 4 training days. (^*^) represents a significant decrease in latency (sec) to find the platform, one way RM ANOVA followed by Bonferroni's *post-hoc*, ^*^P < 0.05, ^***^P < 0.001. groups are represented as (φ) ^φφ^*P* < 0.01, ^φφφ^*P* < 0.001, two way ANOVA. **(D)** This figure shows the latency (sec) of WT (blue) and Fus1 1 KO (red) to find the hidden platform in the NW quadrant over four consecutive trials performed during the four training days. The WT not Fus1 KO mice, were able to reduce their latency over trials; one way RM ANOVA followed by Bonferroni's ^*^P < 0.05, ^***^P < 0.001. Differences between groups were evident over training days, two way ANOVA followed by Bonferroni's *post-hoc*, ^φ^*P* < 0.05, ^φφφ^*P* < 0.001. WT were able to reduce their latency over the training sessions, but the Fus1 KO mice did not show differences between training days.

To know if the Fus1 KO mice have learning and memory impairments we tested them using the Morris water maze task, in which the animals were trained to find the platform quadrant during four consecutive days followed by a final test day without the platform, recording the latency to the correct quadrant. The WT mice reduced their swimming velocity over the training sessions [*R*^2^ = 0.38, *P* < 0.0001, slope −1.5 cm/s/day; *F*_(1.86, 24.17)_ = 7.86, *P* = 0.003, one-way RM ANOVA *N* = 14, Figure 10A] showing a significant reduction between the first day (14.4 ± 0.7 cm/s) compared to the last day of the training (9.5 ± 0.9 cm/s; *P* = 0.005).

Further, the control group also reduced their distance traveled during the training days [*R*^2^ = 0.75, *P* < 0.0001, slope −239.6 cm/day; one-way RM ANOVA *F*_(2.02, 26.32)_ = 39.24, *P* < 0.0001, Figure 10B], showing statistical differences on the first day of the session (1030 ± 85.8 cm) compared with the second day (519.5 ± 71 cm; *P* = 0.0003), third day (336.6 ± 38.3 cm; *P* < 0.0001) and the last day (292.8 ± 38.7 cm; *P* < 0.0001).

Finally, the WT were able to reduce their latency to the platform (*R*^2^ = 0.47, *P* < 0.0001, slope −11.28 s/day; *F*_(1.94, 25.15)_ = 11.54, *P* = 0.0003, one-way RM ANOVA, Figure 10C] showing statistical differences from the first day of training (60.9 ± 4.6 s) compared with the third day (29.9 ± 3.7; *P* = 0.002), and the last day (27.6 ± 3.4 s; *P* = 0.0002).

The velocity observed for the Fus1 KO was also reduced during the session training [*R*^2^ = 0.76, *P* < 0.0001, slope −2.59 cm/s/day, *F*_(2.78, 49.97)_ = 57.72, *P* < 0.0001, one-way RM ANOVA, *N* = 19, Figure 10A], showing a significant reduction from the first day (15.4 ± 0.6 cm/s) compared with the second day (11.2 ± 0.7 cm/s; *P* < 0.0001), the third day (9.03 ± 0.6 cm/s; *P* < 0.0001) and the last session (7.5 ± 0.6 cm/s; *P* < 0.0001). We did not find differences in velocity between WT and KO groups [*F*_(1, 121)_ = 3.14, *P* = 0.08].

The distance traveled by the Fus1 KO animals was also reduced during the sessions (*R*^2^ = 0.61, *P* < 0.0001, slope −294 cm/day, *F*_(2.13, 38.32)_ = 28.32, *P* < 0.0001, one-way RM ANOVA, Figure 10B], and was evident from the first day (1555 ± 124.7 cm) compared with the second day (907.7 ± 124.2 cm; *P* = 0.0006), the third day (726 ± 73.6 cm; *P* < 0.0001) and the last day (635.2 ± 79.7 cm; *P* < 0.0001). However, we found statistical differences between WT and KO groups comparing each day of the training sessions, in which the WT traveled less distance over the training days in comparison to the KO group [*F*_(1, 121)_ = 38.78, *P* < 0.001, two-way ANOVA followed by Bonferroni's *post-hoc P* <0.001 first day, *P* = 0.02 second day, *P* = 0.01 third day, *P* = 0.04 fourth day, Figure 10B].

The most pronounced difference between the WT and Fus1 KO mice was recorded in latency (time from start to goal). Fus1 KO mice did not reduce their latency over the training sessions [*R*^2^ = 0.09, *P* = 0.12; *F*_(2.49, 44.7)_ = 1.8, *P* = 0.17, one-way RM ANOVA, Figure 10C]. We found statistical differences in latency between WT and Fus1 KO groups on all training days [*F*_(1, 124)_ = 70.91, *P* < 0.001, two-way ANOVA, *post-hoc:* first day WT = 60.88 ± 4.7 s vs. Fus1 = 96.6 ± 6.8 s, *P* = 0.006; second day WT = 42.7 ±7 .1 s vs. Fus1 = 80.1 ± 9.4 s, *P* = 0.002; third day WT = 30 ± 3.8 s vs. Fus1 = 83.2 ± 7.6 s, *P* < 0.001; fourth day WT = 27.6 ± 3.4 s vs. Fus1 = 82.5 ± 9.6 s, *P* < 0.001, Figure 10C].

Figure 10D shows the latency of all training trials per day. The WT group reduced their latency across the trials [*R*^2^ = 0.22, *P* < 0.001, slope −2.9 s/trial; *F*_(5.57, 72.37)_ = 3.81, *P* = 0.002, one-way RM ANOVA], showing the first statistical difference in the second trial of the third day (23.8 ± 2.9 s) compared with the first trial on day one (76.8 ± 12.4 s; *P* = 0.01). Unlike WT, the Fus1 KO mice did not show statistical differences in the latency over the trials [*F*_(6.88, 123.9)_ = 0.98, *P* = 0.44].

**Figure 11:**
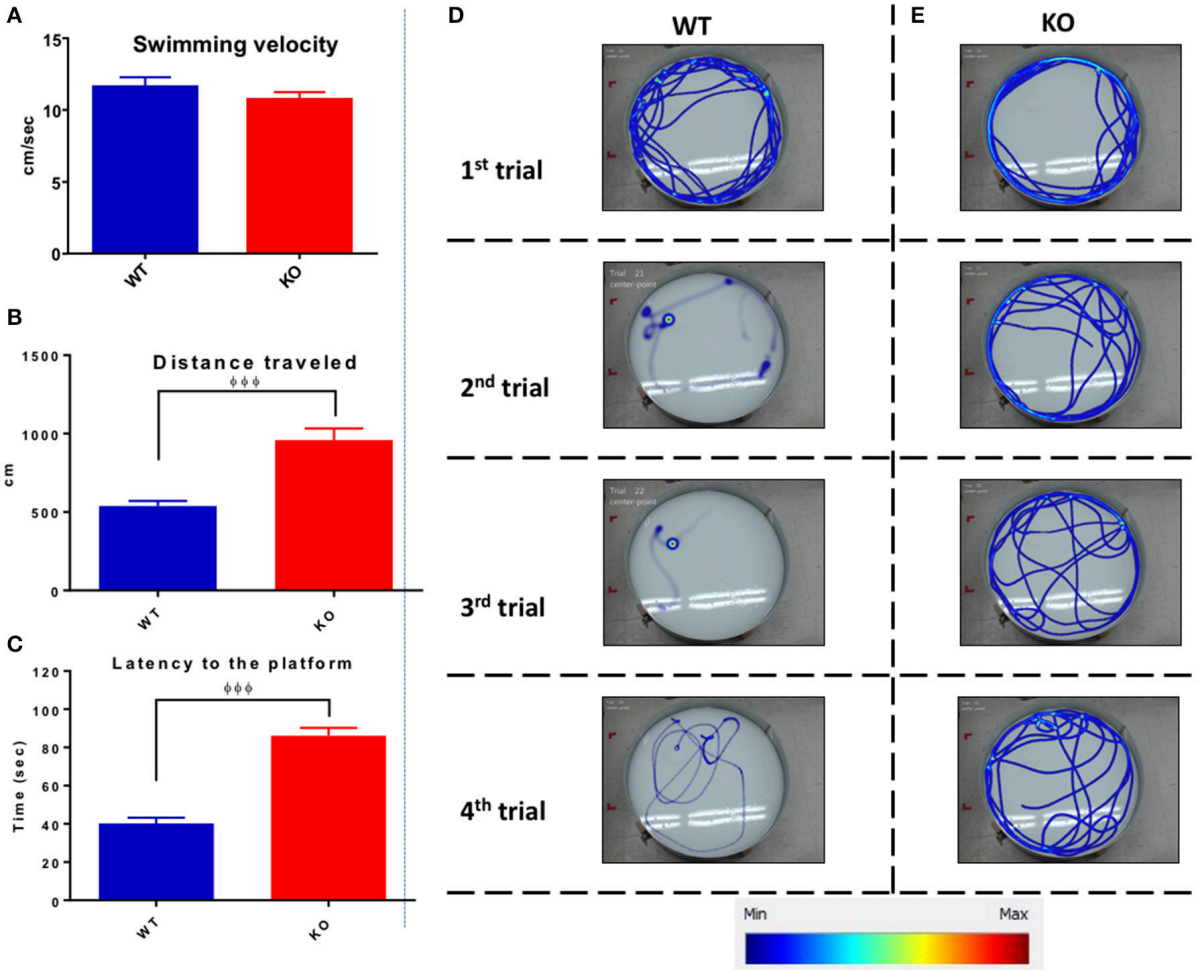
**The average Morris water maze training performance. (A)** The average of swimming velocity (cm/sec) over the 4 days training between WT (blue) and Fus1 1 KO (red). **(B)** The average distance traveled (cm) over the 4 days of training between WT (blue) and Fus1 1 KO (red). Significant group difference was shown using unpaired students *t*-test, ^φφφ^*P* < 0.001. **(C)** The average latency to find the hidden platform performed over 4 training days. The Fus1 KO showed higher latency than the WT group, ^φφφ^*P* < 0.001. **(D,E)** Illustrate representative heat maps of the swimming paths of a WT and Fus1-KO mouse during four trials of the first training day. It is evident that the WT mouse reduced its swimming distance over trials since was able to learn the zone in which the platform was submerged. In contrast, the KO mouse did not decrease its distance and hence did not learn the specific platform zone.

Figure 11 shows the data from the 4 days of training trials illustrating the averaged swimming velocity, traveled distance, and latency for both groups. Average swimming velocity of WT (11.7 ± 0.6 cm/s) and KO (10.8 ± 0.5 cm/s) groups showed no statistical differences between groups (unpaired one-tailed Student's *t*-test *P* = 0.12, Figure 11A). In contrast, Fus1 KO mice traveled on average a longer distance of 958 ± 76 cm than the WT mice (538 ± 34 cm; unpaired one-tailed Student's *t*-test *P* < 0.0001, Figure 11B). Moreover, the Fus1 KO animals spent more time finding the platform (86 ± 4 s) compared with the WT (40.3 ± 3 s; unpaired one-tailed Student's *t*-test *P* < 0.0001, Figure 11C). To illustrate the differences described above, we show the four swimming paths from the first day of training from a WT mouse (Figure 11D) and a KO mouse (Figure 11E). While the WT was able to learn the site of the platform, and therefore decreased its distance traveled and latency to the platform over trials, the Fus1 KO mouse did not show any differences, showing difficulty to learn the spatial memory task.

### Fus1 KO mice showed a low performance in the test day of the MWM task

**Figure 12:**
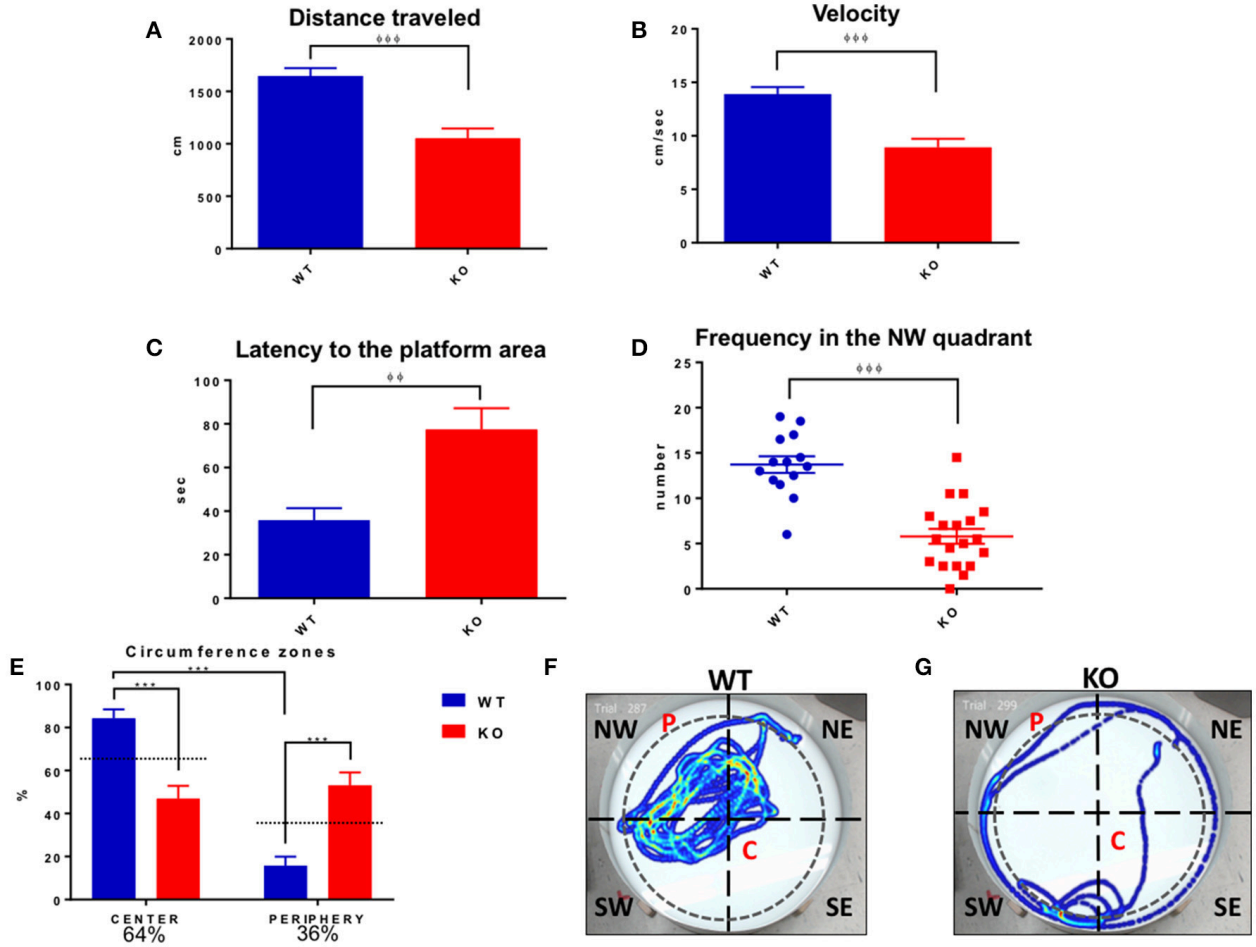
**Morris water maze test day. (A)** Represents distance traveled (cm) by WT (blue) and Fus1 1 KO (red). The WT traveled further than the Fus1 KO, unpaired one-tailed student *t*-test, ^φφφ^*P* < 0.001. **(B)** Shows the reduction in velocity (cm/sec) of the Fus1 KO (red) compared to WT mice (blue) unpaired one-tailed student *t*-test, ^φφφ^*P* < 0.001. **(C)** Illustrates the latency (sec) of WT (blue) and Fus1 1 KO (red) to swim to the platform location in the NW quadrant on test day during two consecutive trials. Fus1 KO displayed greater latency to the area unpaired one-tailed student *t*-test, ^φφ^*P* < 0.01. **(D)** Dot plot showing the number of times the WT crossed into the NW quadrant compared to the KO mice (the zone in which the platform was submerged during the training sessions). Unpaired one-tailed students *t*-test, ^φφφ^*P* < 0.001. **(E)** Depicts the total time (expressed as percentage) that the groups of mice spent in the center or periphery of the water maze on the last day of the test. The WT group spent more time in the center compared to the Fus1 KO, two-way ANOVA followed by Bonferroni's *post-hoc*, ^***^P < 0.0001. **(F,G)** Show heat maps of the swimming path of two mice, WT and Fus1 KO, on the fifth day of the MWM test without the platform. Note the swimming path of the WT mouse that shows frequent visits to the NW quadrant; in contrast the Fus1 KO mouse path does not show a clear preference for the NW quadrant and has less complex movements, suggesting disorientation.

On the test day (locating a quadrant without platform), the Fus1 KO traveled a shorter distance (1053 ± 93.2 cm) than the control group (1648 ± 75.7 cm; unpaired one-tailed Student's *t*-test *P* < 0.0001, Figure 12A). The velocity of the KO (8.9 ± 0.08 cm/s) was less than the background (13.91 ± 0.6 cm/s; unpaired one-tailed Student's *t*-test *P* < 0.0001, Figure 12B). Additionally, the latency to the platform quadrant of the Fus1 KO of 77.4 ± 9.7 s was greater than that of the WT (35.7 ± 5.6 s; unpaired one-tailed Student's *t*-test *P* = 0.001, Figure 12C). Further, the WT group visited the NW quadrant significantly more frequently (13.7 ± 0.9) than the Fus1 group (5.8 ± 0.8; unpaired one-tailed Student's *t*-test *P* < 0.0001, Figure 12D).

Inspection of the paths suggested that the Fus1 KO mice showed thigmotaxis (“wall-hugging”) during the last day of the task. To analyze this, we divided the maze (91 cm ID, 4163 cm^2^) into a center with 80% of the total ID (72.8 cm ID, 4163 cm^2^, 64% of total area) and remainder as the periphery (36% of total maze area). We found that the Fus1 mice spent less time in the center of the maze (46.9 ± 6%) compared with the WT [84.2 ± 4.2%; two-way ANOVA, *F*_(1, 62)_ = 45.15, *P* < 0.0001, followed by Bonferroni's *post-hoc P* <0.0001]. Further, the Fus1 group spent more time in the periphery of the maze (53.1 ± 6%) compared with the WT (15.8 ± 4.2%, *P* < 0.0001). Further, the WT spent less time in the periphery than expected by chance (*P* < 0.001; one-tailed paired *t*-test vs. 36%), while the KO mice spent more time in the periphery than expected by chance (*P* < 0.006, Figure 12E). This observation bears out the KO's impairments to remember a specific location since the platform was always inside the center zone during the training days. To illustrate the above, we show a swimming path from a WT mouse (Figure 12F) which spent more time in the NW quadrant and in the center of the maze, trying to find the platform (P). On the other hand, the KO mouse looked disoriented (Figure 12G), with a simple thigmotaxic navigation path, with no evident sign to remember the location of the P, and spending more time in the periphery of the maze. Interestingly, thigmotaxis was not evident in the open field test for either group. In conclusion, the Fus1 KO mice showed clear evidence of impaired spatial memory.

### Fus1 KO mice did not show alterations in the nestlet building, but did show sleep/awake disturbances compared to the WT group

**Figure 13:**
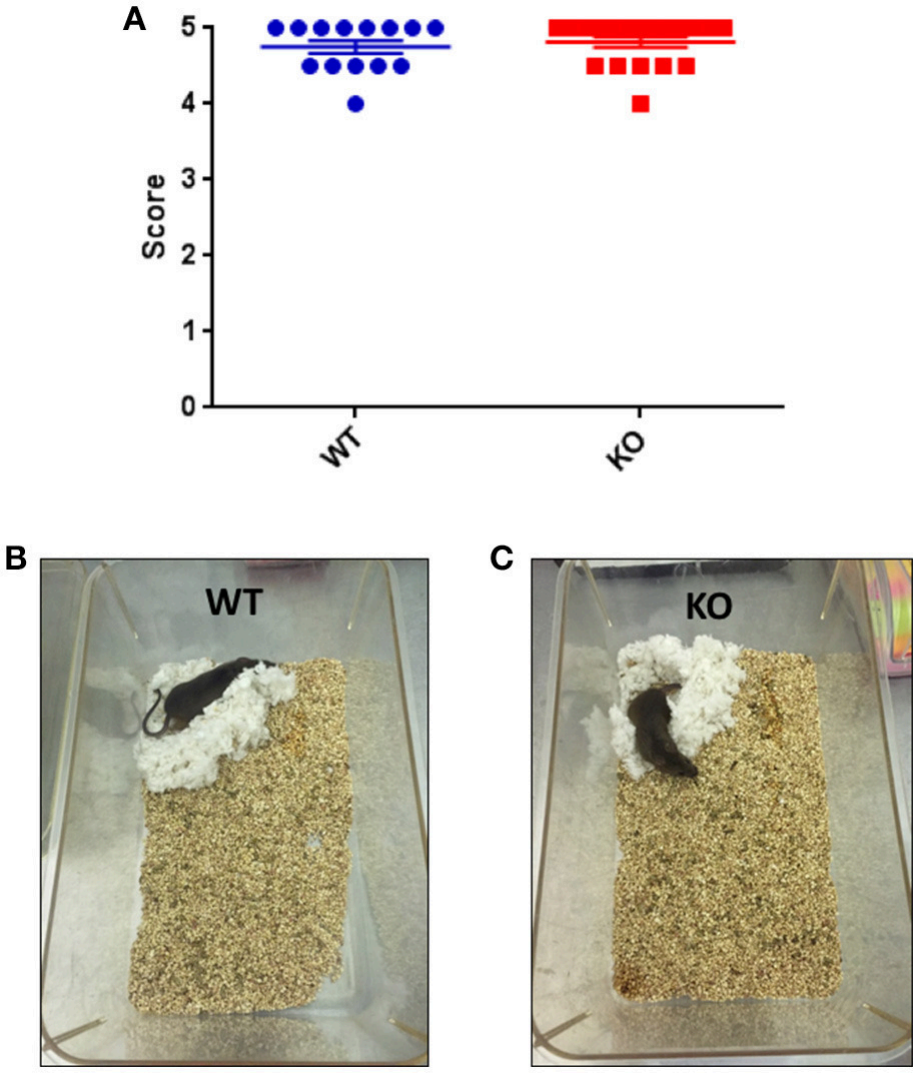
**(A)** Nestlet building test. Scores (Goate et al., 1991; Levy-Lahad et al., 1995; Sherrington et al., 1995; Piaceri et al., 2013; Kumar et al., 2015) defining the construction of nestlet, 1 is an intact nestlet and 5 indicates a nestlet torn over 90% and with a clear crater. WT and Fus1 KO were able to build the nest and did so with no significant difference in their total score. **(B,C)** Show the similar nest building capacities of a WT and a Fus1 KO mouse, showing well-defined nestlet craters.

The nestlet building test measures the capacity of the mice to construct a nest. Both WT and KO mice scored similarly high (4.8 ± 0.09 vs. 4.8 ± 0.07) on this test without statistical difference (unpaired one-tailed Student's *t*-test *P* = 0.28, WT *N* = 14, Fus1 KO *N* = 19, Figure 13A). We show in Figures 13B,C examples of nest building by a WT and KO mouse after 24 h at the end of the test we did not find any differences between groups.

**Figure 14:**
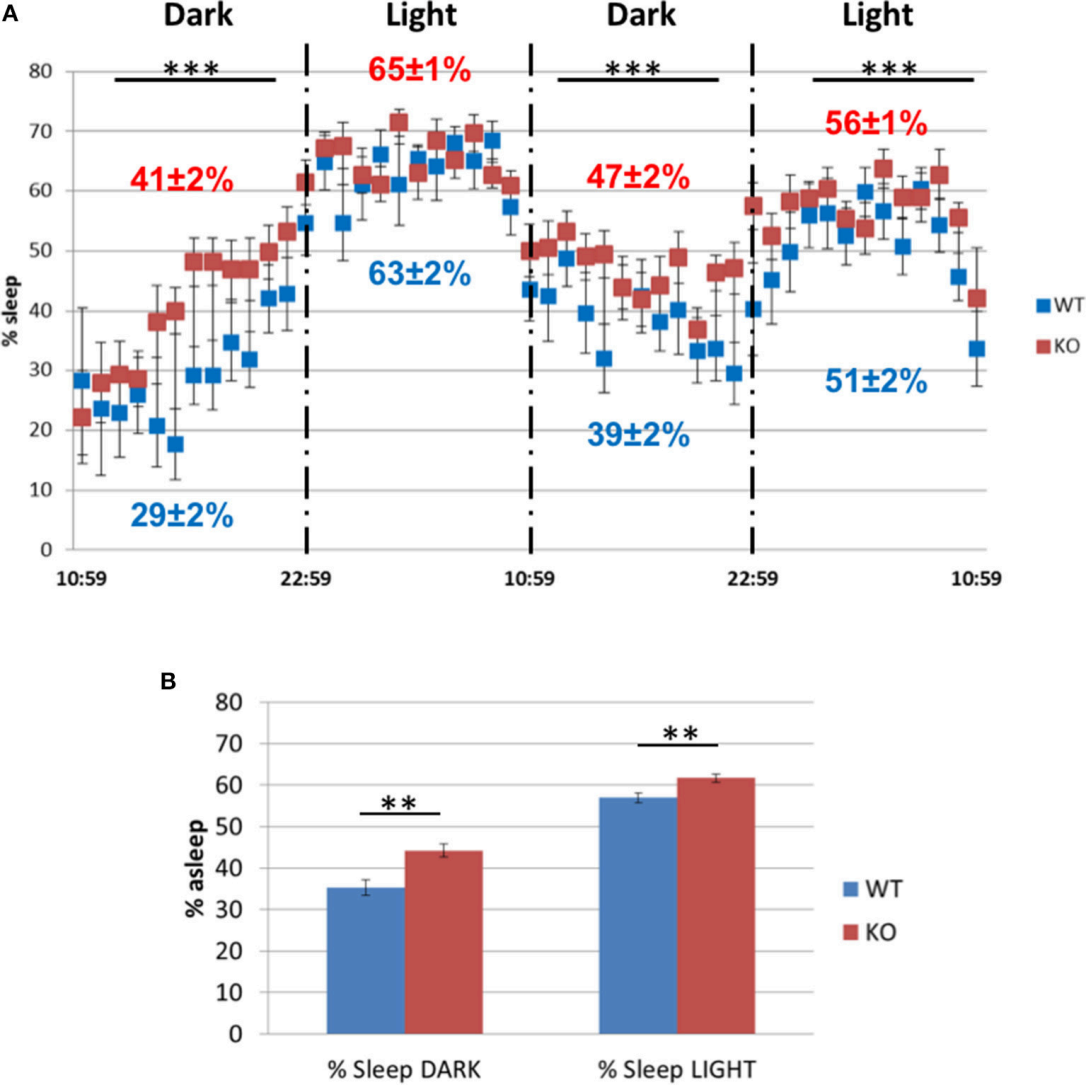
**The sleep/awake test. (A)** Comparison of the percentage of sleep observed in the WT and Fus1 KO mice during two dark and light cycles. The Fus1 KO displayed a higher percentage of sleep compared to the WT. **(B)** Shows the percentage of time the animals were asleep during 2 days for each cycle, which differed between groups during both the dark and light cycles. Paired one-tailed students *t*-test, ^**^P < 0.01, ^***^P < 0.001.

However, in the sleep/awake test during two dark and light cycles (Figure 14A), the Fus1 KO spent a larger fraction of time asleep during the dark cycle of the first day (41 ± 2% asleep vs. WT 29 ± 2%, unpaired one-tailed Student's *t*-test *P* = 0.0001) and the second day (47 ± 2% asleep vs. WT 39 ± 2%, unpaired one-tailed Student's *t*-test *P* = 0.003), and in the light cycle of the second day (56 ± 1% vs. WT 51 ± 2%, unpaired one-tailed Student's *t*-test *P* = 0.007). With respect to the time spent asleep across the 2 days (Figure 14B), we found statistical differences in the dark cycle (WT 35.3 ± 1.9% asleep vs. Fus1 KO 44.3 ± 1.6%, unpaired one-tailed Student's *t*-test *P* = 0.001), and in the light cycle (WT 56.9 ± 1.3% asleep vs. Fus1 KO 61.7 ± 0.9%, unpaired one-tailed Student's *t*-test *P* = 0.003).

### Fus1 KO mice show alterations in energy homeostasis, antioxidant, autophagy, PKC and calcium signaling proteins in the olfactory bulb and (or) hippocampus

In order to comprehend the molecular basis of early alterations in olfactory memory, spatial memory, and sleep commonly associated with aging but identified in 5 months old Fus1 KO mice, we performed immunoblot analysis of olfactory bulbs (OB) and hippocampi (HP) focusing on aging-associated pathways. Equal amounts of OB or HP protein lysates extracted from four mice per group were pooled together to obtain sufficient amounts of protein for multiple immunoblots and to compensate for possible inter-individual heterogeneity within a group. Moreover, to make the analysis even stricter, only changes that showed more than 30% difference between WT and KO samples were taken into consideration. The most pronounced difference that we identified in our analysis were changes in the AMPK (5′ AMP-activated protein kinase)/ACC (Acetyl CoA carboxylase) pathway, which regulates cellular energy homeostasis. We showed that both OB and HP of 5 months old KO mice have lower levels of ACC2 protein (9.1- and 2.38-fold decrease, respectively). ACC2 is an enzyme that produces malonyl-CoA molecule, which negatively regulates the β-oxidation of fatty acids. Thus, decreased level of ACC2 can lead to an increased use of fatty acids for energy (ATP) production, which may result in several negative consequences for brain functioning that will be discussed later. At the same time, we found that ACC1, an enzyme involved in fatty acid synthesis and usually present only in lipogenic tissues in contrast to ACC2, which is predominant in oxidative tissues (Kreuz et al., 2009), is drastically increased in Fus1 KO OBs (16.7-fold increase relative to WT) suggesting an increased need for fatty acids in Fus1 KO OBs. Activated AMPK is the negative regulator of ACC, thus we measured the phosphorylation levels of AMPK (phospho-index, a ratio of phosphorylated and total proteins). Both Fus1 KO OB and HP tissues have higher levels of AMPK phosphorylation (2.17- and 14.3-fold increase in P-index, respectively), which suggest decreased activities of ACC in both tissues.

We also found that antioxidant protection of OB and HP tissues in KO mice is deficient based on the low levels of PRDX1, one of the main antioxidant enzymes (2.46- and 4.73-fold decrease, respectively). The autophagy marker LC3-II showed a decrease in both tissues (1.83-fold decrease in OB and 3.27-fold decrease in HP), pointing to an early onset of autophagy dysregulation in Fus1 KO mice. We did not find differences in the apoptotic marker Bcl-xL between WT and Fus1 KO tissues.

We also analyzed the levels of four proteins representing pathways involved in memory formation, synaptogenesis and aging-associated memory loss. TrkB (BDNF receptor) and NGFR (p75NTR) showed no differences, however, Calretinin and RACK1 levels were lower in the HP of Fus1 KO mice (3.83- and 1.58-fold decrease, respectively), though no differences were observed in OB tissues. The significance of these findings is presented in the Discussion section below.

## Discussion

The majority of current animal models of AD express human genes containing mutations associated with familial AD (fAD). fAD is not common, representing only the 5% of AD cases (Irvine et al., 2008). In contrast, the sporadic AD (sAD) represents 95% of the remaining AD population. However, creating models for sAD has been more challenging due to the complex factors (genetics, physiology, age, diet, physical activity, exposure to environmental hazards, etc.) that interact to cause sAD (Onos et al., 2016). It is well known that age is a high risk factor for developing sAD (Gatz et al., 2006). Aging is itself related to the production of free radicals and it has been proposed that excess of free radicals may contribute to amyloid beta aggregation (Christen, 2000). In the present work, we introduce the Fus1 KO mouse as a novel model for sAD that combines premature aging, chronic oxidative stress due to high ROS production and inadequate anti-oxidant machinery, mitochondrial dysfunction, low grade chronic inflammation and aberrant acute inflammatory response (Ivanova et al., 2007; Uzhachenko et al., 2012, 2014; Hood et al., 2013). This model is highly relevant to sAD as increased ROS generation and oxidative damage occur early in the progress of sAD. In fact, oxidative stress may be involved in the pathogenesis of most neurodegenerative disorders (Gandhi and Abramov, 2012). Taken together, the alleviation of oxidative stress represents a potential therapeutic approach for slowing sAD (Di Matteo and Esposito, 2003). Here, we have demonstrated that relatively young Fus1 KO mice develop primary aging-associated alterations related to olfactory memory, spatial memory and sleep.

Ranging from Warner's smell identification studies testing patients at different stages of disease (Warner et al., 1986), to the olfactory experiments testing transgenic animal models for AD (Coronas-Sámano et al., 2014; Roddick et al., 2016), it has been proposed that there is a decrement in the ability to identify, discriminate and remember a wide variety of odorants. Interestingly, while Fus1 KO animals showed somewhat poor long-term odor memory compared to their WT counterparts, short-term memory was not clearly adversely affected. Two different mechanisms govern habituation memory in mice across the two different timescales (McNamara et al., 2008; Wilson and Linster, 2008; Freedman et al., 2013). The habituation to novel olfactory stimuli is preceded by a complex process of odor recognition whereby olfactory sensory neurons within the nose form glutamatergic synapses on mitral cells which project into the olfactory cortex, principally the piriform cortex, then to the basolateral amygdala. These mitral cells are themselves glutamatergic and target pyramidal cells that express N-methyl d-aspartate (NMDA) and non NMDA metabotropic receptors (Shipley and Ennis, 1996). Antagonists of the metabotropic receptor have been shown to reduce odor habituation via mitral cell projections to the piriform cortex for short term behavioral odor adaptation. In contrast, long term habituation is localized within the olfactory bulb is affected by the NMDA antagonist MK-801 either by systemic injection or introduced locally to the olfactory bulb (McNamara et al., 2008). Therefore, odor habituation over short ITIs is mediated by synaptic adaptation in the piriform cortex by the activation of metabotropic glutamate receptors, which has been shown to persist briefly (ca. 2 min) and is highly odor specific. In contrast, habituation over longer ITIs is dependent on the NMDA receptor, is localized primarily within the bulb, lasts for at least 20 min, and is not odor specific (McNamara et al., 2008). Habituation persistence studies across either short or long time scales have also shown increased exploration time for as long as 60 min post-presentation of an odor (Freedman et al., 2013). Therefore, the differences shown between habituation over short and long time frames in Fus1 KO mice suggests an underlying Fus1-dependent alteration in the habituation mechanism within the olfactory bulb. Fus1 is highly expressed with the olfactory bulb (http://biogps.org/#goto=genereport&id=80385) and within the cerebral cortex which hence does not explain the moderate differences between the short and long term memory defects observed at this young age. The ability to perform the STM task suggests that it is not an alteration in perception of odor but rather a reduction in memory duration as the ITI's were increased. The formation and expression of habituation memory from the olfactory bulb is modulated by noradrenergic projections from the locus coeruleus (LC). Studies have shown that lesioning cortical noradrenergic fibers can eliminate habituation to the same odors, which can be rescued by the infusion of noradrenalin into the olfactory bulbs (Guérin et al., 2008). Noradrenalin has been shown to drastically reduce levels of reactive oxygen species suggesting that it acts as an antioxidant. It is rapidly degraded in this process as it is primarily protective via its metabolites (Troadec et al., 2001). This suggests that within our animal model the excessive ROS production may be decreasing OB noradrenergic levels which are important for habituation memory.

Yang and Crawley illustrate the animal's natural tendency to use olfactory cues during buried food tests that can be used to evaluate their ability to smell volatile odors, assuming that if mice fail to locate the food, they are likely to have deficits in their olfactory abilities (Yang and Crawley, 2009). Moreover, Kulkarni et al. (2012) hypothesized that evolution favored the olfactory system and its connections to the hippocampus and limbic cortex providing rodents a guide to resources of calorie rich food in their environment. This affects multiple areas besides the primary olfactory system, including areas associated with learning and memory and the processing of emotional stimuli: forebrain cortex, insular cortex, hippocampus, nucleus accumbens, anterior thalamic nucleus (Kulkarni et al., 2012). Here we have shown that the Fus1 KO animals can perform the hidden cookie test over a short time frame, however, when these animals are tested again 24 h later no improvement in their performance was observed unlike the WT animals, which displayed a decrease in time taken to find the food reward. The difficulty of Fus1 KO animals to perform the task, however, does not reflect a motivational deficit, as they did not show alterations in the nest building test, whereby high levels of motivation are required to construct an evolutionary beneficial living environment (Jirkof, 2014). They also were not different from WT mice in the sucrose preference test, which is indicative of anhedonia. Experiments mapping post-training memory consolidation during an odor–reward association task, illustrated the involvement of the amygdala and the prefrontal cortex (PFC), both of which also highly express Fus1 (http://biogps.org/#goto=genereport&id=80385). The amygdala has been shown to be important during odor discrimination tasks in concert with the PFC (Schoenbaum et al., 1999). Furthermore, the role of the PFC for long-term memory formation has also been demonstrated during food odor association tasks (Bouret and Sara, 2004; Tronel et al., 2004; Carballo-Márquez et al., 2007). In addition, the important role of the amygdala in inhibitory avoidance learning has been widely demonstrated (Wilensky et al., 2000; Canal et al., 2008). Interestingly, our results demonstrate defects during the odor reward tasks but not during inhibitory avoidance. These results show that despite the overlapping brain regions involved in the olfactory based memory tasks and association memory tasks, defects are only shown for tasks requiring olfaction. Taken together, our results suggest that the mitochondrial dysfunction-mediated increased levels of ROS and low-grade inflammation in young Fus1 KO model of the olfactory-based deficits correlate with precognitive decline associated with sAD.

Long term stress can contribute significantly to cognitive impairment. Hence, emotional symptoms such as anxiety and depression (anhedonia) were other key parameters for investigation as they contribute significantly to the clinical profile in mild cognitive impairment and AD. There was no clear evidence during any test investigating emotional disturbances that Fus1-deficent mice display these symptoms, except for significantly increased latency to cross from the light to the dark box at the beginning of the experiment. This difference in time on crossing from the light box to an unfamiliar environment (dark box) could be a first sign of neophobia (initial tendency to avoid a novel environment) (Bourin and Hascoët, 2003).

Fus1-deficent animals display deficits in spatial memory observed by poor performance during the Morris water maze compared to WT counterparts. The MWM has been frequently utilized to determine cognitive deficits in AD animal models (Bromley-Brits et al., 2011; Baeta-Corral and Gimenez-Llort, 2015), and it has been useful to study hippocampal-dependent learning including short- and long-term spatial memory. Indeed, mice with hippocampal lesions cannot perform the task. Here, we showed that the Fus1 mice were impaired to learn the specific zone of the maze in which the platform was hidden. The last was evident when the Fus1 mice were not able to decrease their latency to find the platform during four subsequent trials over the training days. When we compared the swimming paths between groups, the KO pathways tended to be less complex compared to the background.

**Figure 15:**
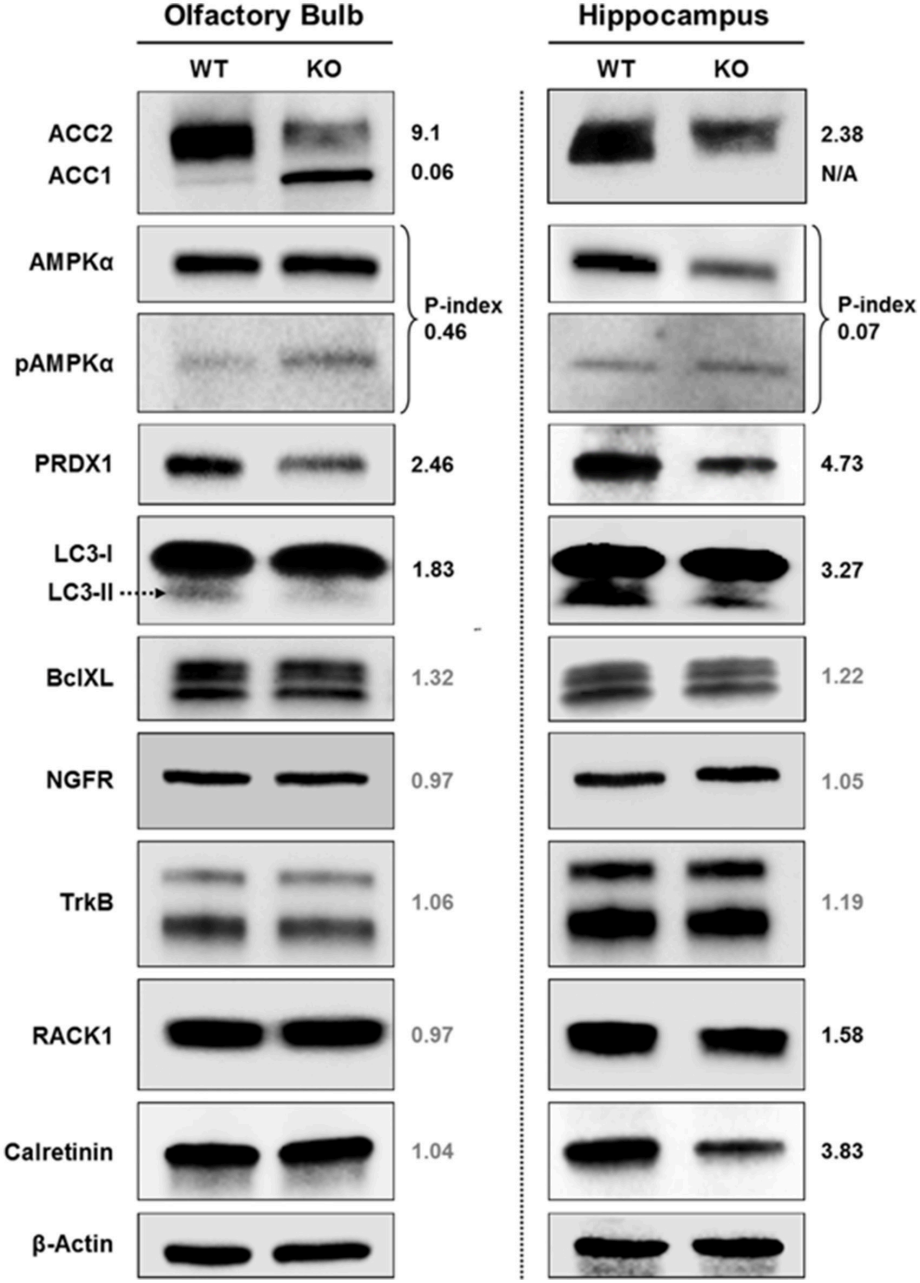
**Western blot analysis of aging-related proteins in the olfactory bulbs and hippocampi of 5-month old WT and Fus1 KO mice**. Proteins involved in energy homeostasis, antioxidant machinery, autophagy, apoptosis, and brain activities were analyzed in the olfactory bulbs and hippocampi of 5-month old WT and Fus1 KO mice by Western blot and are presented as a relative expression or phosphorylation index. The numbers to the right of each western blot panel represent the fold change in protein expression in the WT relative to the KO. All band intensity levels were normalized to a corresponding β-actin level. Phosphorylation index (P-index) is the ratio of phosphorylated protein intensity over total protein band intensity. Only changes that showed more than 30% difference between WT and KO samples were taken into consideration and shown as numbers in black as opposed to numbers in gray.

Moreover, deficits in hippocampal learning and memory have been shown to be linked to oxidative stress associated with aging (Huang et al., 2015). ROS have been shown to adversely affect synaptic plasticity (reviewed in Massaad and Klann, 2011). Since Fus1 is highly expressed in the hippocampus, its loss could result in severe hippocampal oxidative stress (high ROS level and low antioxidant PRDX1 level, Figure 15), thus adversely affecting spatial memory in Fus1 KO mice. The MWM has been frequently utilized to determine cognitive deficits in AD animal models (Sun et al., 2006; Qing et al., 2008). It has been previously demonstrated that aged rats have increased latency to learn the MWM compared to young animals and have decreased retention for the task learned. In addition, young rats fed a vitamin E deficient diet, which results in a defect in their antioxidant capacity, also display inadequate task performance and memory retention compared to control rats of the same age (Fukui et al., 2001). In addition, animals subjected to hypoxia-induced oxidative stress show a reduction in the MWM performance in both young and old mice, which could be alleviated with vitamin E supplementation (Fukui et al., 2002). Changes in diet leading to iron deficiency and dysregulation of manganese has also shown increased oxidative stress markers leading to behavioral deficits in the MWM (Fitsanakis et al., 2009). As discussed, the hippocampus and the olfactory system are required to facilitate the guidance of animals toward food sources. Therefore, the clear deficit observed in hippocampus-dependent tasks suggests that hippocampus may have also played a role in the deficits observed in the hidden cookie task presented in the Fus1 KO mice.

Finally, sleep is critical for the proper function of many organ systems, particularly the brain. The sleep-awake cycle synchronized to the light-dark cycle is the most obvious example of circadian process. Studies suggest that sleep and circadian disturbances likely occur very early in the disease process and may contribute to the pathogenesis of AD. The association between sleep and learning including olfactory-based learning has been demonstrated (Rasch et al., 2007; Barnes and Wilson, 2014). Certain stages of sleep that can be divided into slow wave sleep (SWS), rapid eye movement (REM) and non-rapid eye movement (nREM) have been illustrated to play different roles in memory consolidation during different types of memory tasks (Walker and Stickgold, 2004; Rauchs et al., 2005). Changes in sleep patterns were evident during this study as Fus1 KO mice displayed an increase in the amount of sleep regardless of the light or dark cycle compared to WT animals. However, this increase in sleep time does not appear to be beneficial for the memory-based task performance presented here. Hence it remains to be determined whether these mice have altered sleep stages, which may cause the changes in memory consolidation.

To pinpoint the molecular basis of cognitive alterations in young Fus1 KO mice, we analyzed the primary brain regions involved in olfactory memory; olfactory bulbs and hippocampus. The olfactory bulb processes the olfactory stimuli which then projects to the hippocampus a key area for learning and memory formation. Based on the biology of the Fus1 protein (Uzhachenko et al., 2012, 2014, 2015; Yazlovitskaya et al., 2013, 2015) and on aging-associated pathways involved in cognitive decline, we included in the analysis the proteins representing pathways linked to (1) energy homeostasis (ACC1, ACC2, AMPK); (2) oxidative stress (PRDX1); (3) apoptosis and autophagy (Bcl-xL, LC3); (4) learning and memory (TrkB, NGFR, Calretinin, Rack1). The most consistent alterations observed in both tissues were of the proteins regulating energy homeostasis and anti-oxidant machinery, allowing us to consider dysregulation of energy homeostasis and oxidative stress as the primary pathological events in Fus1 KO mice.

Significant decrease in the levels of ACC2, a negative regulator of fatty acid β-oxidation, combined with increased phosphorylation index of AMPK, that suppresses activities of ACC2 suggest that Fus1 KO olfactory bulbs (OB) and hippocampus (HP) use fatty acid oxidation for ATP production at a much higher levels than WT tissues. Moreover, consistent with this notion, we showed that Fus1 KO OB also have higher levels of ACC1 protein that is responsible for biosynthesis of fatty acids suggesting higher needs by Fus1 KO olfactory bulbs for fatty acids. Increased fatty acid oxidation, most likely, is a compensatory mechanism for inefficient oxidative phosphorylation machinery in Fus1 KO mitochondria (Uzhachenko et al., 2014, and unpublished data) allowing brain tissues to have sufficient energy to survive and function. However, favoring fatty acids oxidation as a source of energy in Fus1 KO brain tissues could result in several disadvantages considering that in general brain metabolism favors glucose oxidation over burning of fatty acids for energy production (Schonfeld and Reiser, 2013). There are several reasons for that: (1) ATP generation linked to β-oxidation of fatty acids demands more oxygen than glucose, thereby enhancing the risk for neurons to become hypoxic; (2) β-oxidation of fatty acids generates superoxide, which, taken together with the poor anti-oxidative defense in neurons, causes increased oxidative stress. In the case of Fus1 KO, considering perpetually high levels of ROS in these cells due to mitochondrial dysfunction (Uzhachenko et al., 2012, 2014; Yazlovitskaya et al., 2015) and low levels of antioxidant PRDX1 in HP and OB tissues (Figure 15), using fatty acids for ATP production in Fus1 KO brain tissues may create such severe oxidative stress that it would be incompatible with normal tissue functioning and result in multiple disturbances on molecular, cellular and behavioral levels; (3) the rate of ATP generation based on adipose tissue-derived fatty acids is lower than that using blood glucose as fuel. Thus, in periods of extended continuous and rapid neuronal firing, that happens, among other cases, during memory formation, fatty acid oxidation cannot guarantee rapid ATP generation in neurons (Schonfeld and Reiser, 2013), therefore, learning and long term memory may be compromised as we found in Fus1 KO mice.

Interestingly, in both OB and HP of Fus1 KO mice we also observed decreased levels of the autophagy marker LC3-II. Autophagy is responsible for the recycling of long-lived proteins and organelles that are either damaged (e.g., mitochondria) or functionally redundant and is crucial for the maintenance of cellular homeostasis in all eukaryotic cells. The primary role of autophagy is to protect cells against stress (Moreau et al., 2010). During autophagy a cytosolic form of LC3 (LC3-I) is converted via conjugation to phosphatidylethanolamine to LC3-II, which is recruited to autophagosomes. High levels of LC3-II reflect a higher autophagy rate and *vice versa*. Defects in the activation of autophagy are involved in the pathogenesis of AD (Wolfe et al., 2013; Nixon, 2014), thus we suggest that defective autophagy is one of the several of molecular mechanisms playing a detrimental role in early cognitive decline of Fus1 KO mice.

We also found that expression of two proteins, RACK1 (receptor for activated C-kinase 1) and Calretinin (Calb2) is downregulated in HP but not in OB of Fus1 KO animals. RACK1 is an anchoring protein that shuttles activated PKC to cellular membranes, plays an important role in PKC-mediated signal transduction pathways (Mochly-Rosen et al., 1991). It is ubiquitously expressed in the brain, especially at higher levels in memory related brain areas, such as the hippocampus, cortex, and cerebellum (Ashique et al., 2006). It has been reported that expressions of RACK1 were significantly decreased in the brain of aging animals and AD patients (Battaini et al., 1999; Van der Zee et al., 2004). Calretinin (Calb2) is a calcium-binding protein that is expressed principally in neurons (Rogers, 1987). It has an important role as a modulator of neuronal excitability including the induction of long-term potentiation, the basis of learning and memory (Camp and Wijesinghe, 2009). Calretinin is also involved in presynaptic signaling as a mobile calcium buffer/transporter capable of regulating calcium signaling over nanometer distances at presynaptic sites (Edmonds et al., 2000). Calretinin interneurons are early targets of extracellular amyloid-beta pathology in the hippocampus of Alzheimer mouse models (Baglietto-Vargas et al., 2010). Thus, taken the importance of these two proteins in neuronal activities, we suggest that Fus1-dependent decrease in RACK1 and calretinin plays a detrimental role in cognitive activities of these mice.

Interestingly, two other proteins that we analyzed, TrkB and NGFR, which also are closely involved in memory formation and in normal development and survival of neurons in the peripheral and central nervous systems (Vickland et al., 1991; Mizuno et al., 2003), did not show any difference between WT and KO. This suggests that Fus1 loss affects specific neuronal pathways associated with calcium and PKC signaling.

Fus1 affects a wide range of proteins, the mechanism of which is only partially known. Fus1 is a nuclear-encoded, mitochondria-residing small globular protein with a yet unknown biochemical activity. We found that it is a putative calcium-binding protein that regulates mitochondrial and cellular calcium fluxes (Uzhachenko et al., 2014), ROS production (Uzhachenko et al., 2012), mitochondrial dynamics (Uzhachenko et al., 2014), and efficiency of anti-oxidant machinery (Yazlovitskaya et al., 2013, 2015, and present manuscript). Importantly, Fus1 KO cells have low mitochondrial respiratory reserve (manuscript submitted), which means they cannot produce additional ATP/energy in response to stimuli or stress. Thus, loss of Fus1 may alter expression/phosphorylation of genes/proteins that are modulated by calcium, ROS or ATP levels as well as by oxidative stress, which we showed in this study (Figure 15) and our other studies (Ivanova et al., 2007; Hood et al., 2013; Uzhachenko et al., 2014, 2015).

As the olfactory tract projects to the piriform cortex, amygdala, and hippocampus, which are all severely damaged in AD, this has led to the “olfactory hypothesis” of AD. It suggests that the olfactory pathway may be an initial site of involvement in the disease progression (Mann et al., 1988; Kovács et al., 2001). This is confirmed by AD degenerative changes observed within the olfactory bulb, tract and anterior olfactory nucleus (Powell et al., 1963; Pearson et al., 1985). Although sAD and fAD present with clinical phenotypes that are very similar, their molecular mechanisms differ substantially as genetic mutations are not the singular cause of sAD. The reason for amyloid accumulation and aggregation therefore, remains to be fully elucidated. Consequently, transgenic mouse models alone are insufficient to investigate sAD etiology and pathogenesis (Lecanu and Papadopoulos, 2013). Transgenic approaches used to investigate AD rely on the ectopic expression of the mutated human genes, such as APP/PS1 and APP/Tau, that drive fAD but are not representative of the underlying pathology of sAD, which is more prevalent in the population (Borchelt et al., 1997; Lewis et al., 2001). As such approaches only model the amyloidogenesis hypothesis of AD pathology, novel tools are required to investigate sAD *in vivo*.

The large array of tests used here to characterize the Fus1 KO mouse required careful design. In order to minimize the influence of one test on the next and to avoid manipulating the aging process the following features were integrated. First, we avoided tests that require food or water regulation which may affect homeostasis, stress and the aging process. Hence all tests are “spontaneous.” Second, the more stressful tests (MWM and passive avoidance) were implemented near the end of the test set (Figure 1). Third, hypothermic stress of the classic MWM was reduced by elevating the water temperature from room temperature to midway (29°C) between room (21°C) and body temperature (37°C). Fourth, 2 days of rest was provided between tests. This was a compromise between increasing the inter-test interval for increased test independence and decreasing the overall duration of the test set (2 months) to assess behavioral abilities within a narrow age range.

Recently, another promising non-transgenic rodent model for investigating central insulin resistance has been described. This model involves the administration of streptozotocin-intracerebroventricularly (STZ-icv) to rats to induce pathological similarities to those observed in sAD patients. These rats demonstrate cognitive deficits at 2 weeks post-administration and last for 12 weeks or longer irrespective of the animal age at the time of administration. This subsequently results in oxidative stress 1 week after treatment, 2 weeks before the memory deficits were observed (Salkovic-Petrisic and Hoyer, 2007). Insulin resistance both centrally and peripherally is a severe risk factor for sAD. Since the olfactory bulb has the brain's highest Insulin Receptor (IRs) density (Unger et al., 1989) and the fastest insulin transport of any brain region (Banks, 2004), it is the most susceptible to central insulin-resistance. As oxidative stress (Butterfield, 2004), changes in the glucose metabolism, cholinergic system, accumulation of tau and beta-amyloid (Grünblatt et al., 2007), tau hyperphosphorylation, neuroinflammation (Weinstock and Shoham, 2004), and learning and memory dysfunctions are all induced by central insulin resistance, it suggests that the alteration in the OB function is a key early indicator of sAD, which is consistent with the data presented here based on the Fus1 KO mouse model.

It could be argued that the Fus1 KO model, showing clear early life cognitive deficits due to a single genetic event, a loss of Fus1, may not represent sAD's multifactorial long-term etiology. Admittedly, it is a difficult task to distinguish between genetic and other factors playing principal roles in the AD etiology, especially considering that no Fus1 mutational analysis in AD patients were performed to date. However, based on our earlier work (Ivanova et al., 2009; Uzhachenko et al., 2012), which showed that Fus1 mRNA level is decreased during ROS exposure, inflammation, and exposure to infection agents, we suggest that Fus1 levels may be regulated epigenetically. Therefore, during chronic conditions that create oxidative stress such as systemic inflammation, or during chronic exposure to environmental hazards, Fus1 mRNA levels may be permanently low, causing chronic mitochondrial dysfunction and consequently early aging and aging-associated pathologies including cognitive deficit. Considering the principal role of mitochondrial bioenergetics in neurodegenerative diseases including AD (Johri and Beal, 2012), here we demonstrate that loss/decrease of Fus1 may cause olfactory and spatial memory changes early in life, which are paralleled by molecular changes in the functionally relevant olfactory bulb and hippocampus. However, we propose that deleterious changes in mitochondrial function caused by Fus1 loss do not immediately result in neurodegeneration and cognitive deficits. This process is rather cumulative in nature and occurs over time, and therefore analogous to spontaneous AD. It consists of (a) an initial period, when no negative consequences of Fus1 loss are noticed due to, most likely, innate compensatory mechanisms; (b) the middle reversible period (the age is 4–5 months old) when the first signs of neurodegeneration could be revealed, and (c) the final, irreversible period (after 11–12 months of age), that is characterized by severe neurodegeneration and lack of plasticity.

We further note that only a subset of the large behavioral test set showed statistically significant decline, most of them of only mild degree at this relatively young age. This parallels the disease progression of sAD which begins with olfactory deficits followed by onset of mild cognitive impairments. These striking parallels between the Fus1 KO model and sAD lend credit to the mitochondrial cascade hypothesis of sAD. It will hence be of great interest to explore the Fus1 KO deficits into senescence. We also suggest that during the first two aforementioned periods the mitochondrial and neurodegenerative changes may be alleviated/corrected by nutraceutical or pharmaceutical intervention that potentiates mitochondrial bioenergetics. This hypothesis will be addressed in our future studies.

Here we demonstrated clear evidence that our novel Fus1 KO mouse model of mitochondrial dysfunction and oxidative stress can be used for the detection of early olfactory memory deficits and spatial memory deficits in young animals and is an excellent alternative mouse model to the classical transgenic animal models of AD.

## Author contributions

GC, JV, AI conceived and designed the study. GC executed the behavioral studies under supervision of JV, with data analysis and graphing by GC, KB, and JV. WT executed the molecular studies under supervision of AI, with data analysis and graphing by AI. GC, JV, AI, WT, and KB contributed to the writing of manuscript and all take full responsibility for and approve of all its content.

### Conflict of interest statement

The authors declare that the research was conducted in the absence of any commercial or financial relationships that could be construed as a potential conflict of interest.
